# Cost-Effectiveness of a Polypill for Cardiovascular Disease Prevention in an Underserved Population

**DOI:** 10.1001/jamacardio.2024.4812

**Published:** 2025-01-08

**Authors:** Ciaran N. Kohli-Lynch, Andrew E. Moran, Dhruv S. Kazi, Kirsten Bibbins-Domingo, Neil Jordan, Dustin French, Yiyi Zhang, Thomas J. Wang, Brandon K. Bellows

**Affiliations:** 1 Department of Preventive Medicine, Feinberg School of Medicine, Northwestern University, Chicago, Illinois; 2 Division of General Medicine, Columbia University Irving Medical Center, New York, New York; 3 Richard A. and Susan F. Smith Center for Outcomes Research, Beth Israel Deaconess Medical Center, Boston, Massachusetts; 4 Division of Cardiology, Beth Israel Deaconess Medical Center and Harvard Medical School, Boston, Massachusetts; 5 Department of Epidemiology, University of California, San Francisco, San Francisco; 6 Department of Psychiatry & Behavioral Sciences, Feinberg School of Medicine, Northwestern University, Chicago, Illinois; 7 Department of Medical Social Sciences, Feinberg School of Medicine, Northwestern University, Chicago, Illinois; 8 Department of Ophthalmology, Feinberg School of Medicine, Northwestern University, Chicago, Illinois; 9 Veterans Affairs Health Services Research and Development Service, Chicago, Illinois; 10 Department of Internal Medicine, University of Texas Southwestern Medical Center, Dallas

## Abstract

**Importance:**

The Southern Community Cohort Study (SCCS) Polypill Trial showed that a cardiovascular polypill (a single pill containing a statin and 3 half-standard dose antihypertensive medications) effectively controls cardiovascular disease (CVD) risk factors in a majority Black race and low-income population. The cost-effectiveness of polypill treatment in this population has not been previously studied.

**Objective:**

To determine the cost-effectiveness of the cardiovascular polypill.

**Design, Setting, and Participants:**

A discrete-event simulation version of the well-established CVD policy model simulated clinical and economic outcomes of the SCCS Polypill Trial from a health care sector perspective. A time horizon of 10 years was adopted. Polypill treatment was priced at $463 per year in the base-case analysis. Model input data were derived from the National Health and Nutrition Examination Survey, Medical Expenditure Panel Survey, pooled longitudinal cohort studies, the SCCS Polypill Trial, and published literature. Two cohorts were analyzed: an SCCS Polypill Trial–representative cohort of 100 000 individuals and all trial-eligible non-Hispanic Black US adults. Study parameters and model inputs were varied extensively in 1-way and probabilistic sensitivity analysis.

**Exposures:**

Polypill treatment or usual care.

**Main Outcome and Measures:**

Primary outcomes were direct health care costs (US dollar 2023) and quality-adjusted life-years (QALYs), both discounted 3% annually, and the incremental cost per QALY gained.

**Results:**

In the trial-representative cohort of 100 000 individuals (mean [SD] age, 56.9 [5.9] years; 61 807 female [61.8%]), polypill treatment was projected to yield a mean of 1190 (95% uncertainty interval, 287-2159) additional QALYs compared with usual care, at a cost of approximately $10 152 000. Hence, polypill treatment was estimated to cost $8560 per QALY gained compared with usual care and was high value (<$50 000 per QALY gained) in 99% of simulations. Polypill treatment was estimated to be high value when priced at $559 or less per year and cost saving when priced at $443 or less per year. In almost all sensitivity analyses, polypill treatment remained high value. In a secondary analysis of 3 602 427 trial-eligible non-Hispanic Black US adults (mean [SD] age, 55.4 [7.6] years; 2 006 597 female [55.7%]), polypill treatment was high value, with an estimated cost of $13 400 per QALY gained.

**Conclusions and Relevance:**

Results of this economic evaluation suggest that polypill treatment could be a high value intervention for a low-income, majority Black population with limited access to health care services. It could additionally reduce health disparities.

## Key Points

**Question:**

Would a cardiovascular polypill (single pill containing a statin and 3 half-standard dose antihypertensives) be cost-effective vs usual care in a majority Black race and low-income population?

**Findings:**

In this economic evaluation, a computer simulation model was used to assess the cost-effectiveness of a cardiovascular polypill in a cohort of 100 000 individuals, representative of the Southern Community Cohort Study Polypill Trial population, who were probabilistically sampled from 3720 individuals in the National Health and Nutrition Examination Survey. Polypill treatment was projected to cost $8560 per quality-adjusted life-year (QALY) gained compared with usual care and was high value (<$50 000 per QALY gained) in 99% of simulations.

**Meaning:**

Results suggest that polypill treatment would be high value if priced at $463 or less per year and could reduce income-related health disparities.

## Teaser

This economic evaluation investigates the cost-effectiveness of a cardiovascular polypill (single pill containing a statin and 3 half-standard dose antihypertensives) vs usual care in a majority Black race and low-income population.

## Introduction

Non-Hispanic Black and lower-income Americans experience higher cardiovascular disease (CVD) rates than their non-Hispanic White and higher-income peers, respectively.^1^ They also have lower rates of control of high blood pressure (BP) and low-density lipoprotein cholesterol (LDL-C).^2,3^ Disparities in control of these risk factors may be due to varying hypertension rates,^4,5^ clinical inertia and missed physician visits,^6^ constrained access to consistent health care,^2^ and disparities in treatment adherence.^7^ Cardiovascular polypills, which are fixed-dose combinations of proven BP- and LDL-C–lowering medications, can address these barriers and be more effective than traditional CVD prevention using separate pills. Polypill treatment enhances patient adherence, reduces the need for dose intensification, reduces adverse effects, and may reduce racial, ethnic, and income disparities in CVD.^8,9^

The Southern Community Cohort Study (SCCS) Polypill Trial was a randomized clinical trial that recruited individuals living within 50 miles of a federally qualified community health center in Franklin, Alabama.^10^ The trial included 303 adults without CVD (mean [SD] age, 56.0 [6.0] years; 292 Black individuals [96%], race was self-reported by the participant; 182 female [60.1%], and 227 individuals [74.9%] with annual income <$15 000), randomized to receive either a cardiovascular polypill or usual care. The polypill arm received 90-day refillable supplies of a cardiovascular polypill containing half doses of 4 preventive agents (ie, atorvastatin, 10 mg; amlodipine, 2.5 mg; losartan, 25 mg; and hydrochlorothiazide, 12.5 mg). The control arm received routine care at the health center alongside ongoing baseline care. In both treatment arms, clinicians could escalate or deescalate therapy. After 12 months, the polypill arm experienced a mean reduction of 7 mm Hg (95% CI, 2-12 mm Hg) in systolic blood pressure (SBP) and 11 mg/dL (95% CI, 5-18 mg/dL) in LDL-C level (to convert to millimoles per liter, multiply by 0.0259) compared with the control arm. These reductions were comparable with those in other primary prevention polypill trials.^11^

Currently in the US, there are no polypills like the one used in the SCCS Polypill Trial that are commercially available. Health economic analyses are crucial for pricing, implementation, and scale-up of novel clinical interventions.^12^ We estimated the long-term cost-effectiveness of a primary prevention cardiovascular polypill in 2 cohorts: an SCCS Polypill Trial–representative cohort and all non-Hispanic Black US adults meeting the trial eligibility criteria. We also estimated the distributional effect of a polypill intervention on health disparities.

## Methods

The institutional review boards (IRBs) at the Centers for Disease Control and Prevention approved the National Health and Nutrition Examination Survey (NHANES) protocols. All National Heart, Lung, and Blood Institute (NHLBI) cohorts used were approved by IRBs at participating institutions. In all analyzed studies, every participant provided written informed consent. This study followed the Consolidated Health Economic Evaluation Reporting Standards (CHEERS) reporting guidelines (eTable 8 in Supplement 1).

### Simulation Overview

**Figure 1.**
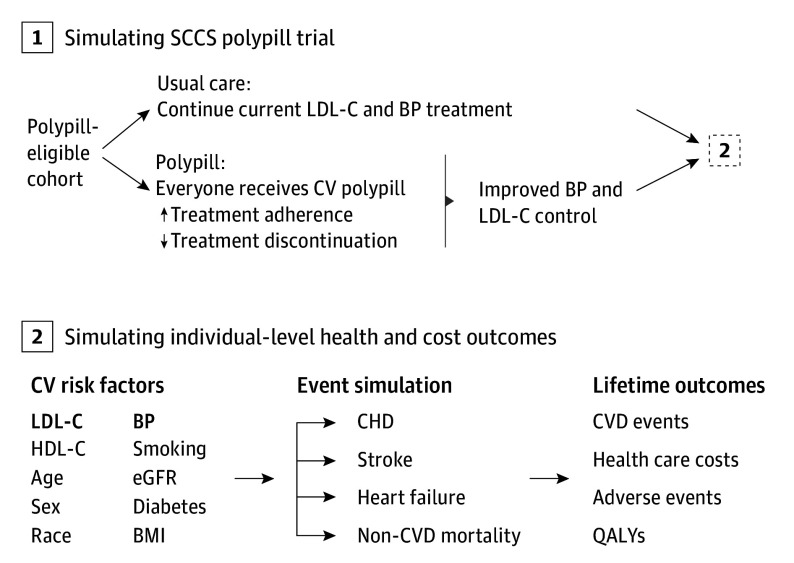
Cardiovascular Disease (CVD) Policy Model, Discrete Event Simulation Version A subset of individuals was constructed with risk factor profiles resembling the Southern Community Cohort Study (SCCS) Polypill Trial population. Each individual was simulated 2 times, receiving: (1) usual care (continuation of current low-density lipoprotein cholesterol [LDL-C] and blood pressure [BP] treatment) and (2) the cardiovascular polypill (containing atorvastatin, 10 mg; amlodipine, 2.5 mg; losartan, 25 mg; and hydrochlorothiazide, 12.5 mg). Polypill-treated individuals had improved adherence, lower rates of discontinuation, and better LDL-C and BP control than those receiving usual care. The CVD policy model predicted risk of coronary heart disease (CHD), stroke, heart failure, and non-CVD mortality, dependent on individuals’ CVD risk factors. An individual may experience more than one nonfatal event. The model calculates lifetime health and cost outcomes as determined by these events. BMI indicates body mass index; eGFR, estimated glomerular filtration rate; HDL-C, high-density lipoprotein cholesterol; QALY, quality-adjusted life-year.

A discrete event simulation version of the well-established CVD Policy Model (CVDPM) simulated clinical and economic outcomes of the SCCS Polypill Trial (Figure 1).^13,14,15,16^ The CVDPM simulates the health care processes of CVD prevention and management (ie, physician visits, measurement accuracy, probability of medication initiation and intensification, and medication adherence) to project BP and LDL-C changes, fatal and nonfatal CVD events, survival, quality-adjusted survival, and direct health care costs. The events in the simulation are (1) physician office visits (related to BP and LDL-C screening and treatment), (2) medication-related adverse events, (3) medication discontinuation, (4) fatal or nonfatal CVD events, and (5) non-CVD death. Included CVD events are coronary heart disease (CHD; ie, myocardial infarction, cardiac arrest, and other coronary heart disease), stroke, and incident heart failure.

### Simulated Populations

The CVDPM simulates a nationally representative cohort of individuals from NHANES 1999 through 2018 who have previously had life-course trajectories imputed for their CVD risk factors (ie, LDL-C, high-density lipoprotein cholesterol [HDL-C], total cholesterol, SBP, diastolic blood pressure [DBP], body mass index [BMI], smoking status, estimated glomerular filtration rate [eGFR], and diabetes status).^17,18^ For this analysis, we included individuals who met the inclusion and exclusion criteria of the SCCS Polypill Trial (eFigure 1 in Supplement 1). Trial inclusion criteria were ages 40 to 75 years, SBP of 120 to 160 mm Hg, LDL-C level less than 190 mg/dL, current use of 2 or fewer BP-lowering medications, and no reported history of CVD, cancer, liver disease, or insulin-dependent diabetes.

Typically, the CVDPM uses probabilistic sample weights to simulate the US adult population. In the base case, we reweighted participants to create a cohort that was representative of the SCCS Polypill Trial population (eMethods, eTable 1 in Supplement 1). Specifically, we reweighted the population to replicate the trial’s baseline SBP, DBP, LDL-C level, HDL-C level, BMI, age, sex, race, statin use, antihypertensive medication use, and annual household income. In a secondary analysis, we simulated scaling the cardiovascular polypill up to a nationally representative population of non-Hispanic Black US adults who met the trial eligibility criteria (eFigure 1 in Supplement 1). For this analysis, we projected the costs and benefits of the polypill across the estimated 3.6 million non-Hispanic Black US adults meeting the eligibility criteria. We also calculated the distribution of expected health gains from polypill therapy by poverty to income ratio in this population.

### Intervention and Comparator

The simulation commences with a physician visit, at which a patient receives a cardiovascular polypill or continues usual care. In the polypill arm, all individuals commenced polypill treatment. Those receiving other antihypertensive and lipid-lowering treatments at baseline switched to the polypill. Individuals who discontinued the polypill reverted to usual care.

Individuals with baseline medication in the usual care arm continued this treatment. Subsequent treatment was initiated dependent on BP level, LDL-C level, and 10-year Atherosclerotic CVD (ASCVD) Risk Score at physician visits.^19,20^ Individuals with uncontrolled BP (SBP ≥130 mm Hg or DBP ≥80 mm Hg) were considered for antihypertensive medication, as recommended by the American College of Cardiology/American Heart Association (ACC/AHA) BP guideline.^21^ Individuals with LDL-C level of 190 mg/dL or higher, diabetes, ASCVD Risk Score of 7.5% or higher, or ASCVD Risk Score from 5.0% to 7.5% with LDL-C level of 160 mg/dL or higher were eligible for statin therapy, as recommended by the ACC/AHA lipid guideline.^20^ Probability of treatment intensification (ie, new treatment initiation or titration) was calibrated to replicate 1-year results from the polypill trial; 10% of participants in the usual care arm experienced treatment intensification during follow-up. Probability of commencing statin therapy when eligible was equal to the proportion of eligible patients in the simulation cohort who were receiving statin therapy at baseline.

### Outcomes

We projected the costs and benefits associated with the polypill vs usual care over a 10-year time horizon, the maximum anticipated time that the polypill could be prescribed to individuals with BP of 130/80 mm Hg or higher without requiring treatment intensification. A health care sector perspective was adopted, including all direct medical costs. Primary outcomes were health care costs (2023 US), quality-adjusted life-years (QALYs), and the incremental cost-effectiveness ratio (ICER). Future costs and QALYs were discounted 3.0% annually.^22^ All costs were inflated to 2023 US dollar from their baseline year using the consumer price index for medical care.^23^ A strategy was considered high value if its ICER was less than $50 000 per QALY gained, intermediate value if its ICER was $50 000 to $150 000 per QALY gained, and low value if its ICER was greater than or equal to $150 000 per QALY gained.^24^ Secondary outcomes were CVD events, life-years, and physician visits. We also recorded disaggregated costs (ie, medication, acute CVD, chronic CVD, non-CVD health care) and QALYs (ie, acute CVD, chronic CVD, background, treatment disutility).

### Model Inputs

Time between physician visits was derived from the Medical Expenditure Panel Survey (MEPS) 2017 and varied based on characteristics including baseline medication status, BP increase since last visit, treatment intensification at last visit, and age (eTable 2 in Supplement 1).^19^ Patients with controlled BP required less frequent visits than those with uncontrolled BP. Time to incident CVD events and non-CVD mortality were estimated using Cox proportional hazards models derived in the NHLBI Pooled Cohort Study (NHLBI-PCS); details are available in eTable 3 in Supplement 1. Data on recurrent events, revascularization, and case fatality rates came from published literature and national surveys.^13,14,15,18^ Cost and health state utility values were derived from published literature and national data sources (eMethods and eTable 4 in Supplement 1).

Medication effects were derived from published studies and other publicly available data (Table 1 and eMethods and eTables 5-7 in Supplement 1).^10,19,22,23,25,26,27,28,29,30,31,32,33,34,35,36,37,38,39,40,41,42,43^ Change in SBP attributable to medication was estimated from published meta-analyses and calculated as a function of dosage and pretreatment SBP.^26,27,44^ Reductions in SBP lowered CVD event risk, as observed in a meta-analysis of randomized clinical trials.^36^ Relative risks of incident CHD, stroke, and non-ischemic HF per 10 mm Hg reduction in SBP were 0.83, 0.73, and 0.72, respectively.^36^ Percentage reduction in untreated LDL-C attributable to intermediate-intensity statin therapy was 37%.^28^ Relative risks of incident CHD and stroke per 38.67 mg/dL (1.0 mmol/L) reduction in LDL-C were 0.76 and 0.85, respectively.^40^

**Table 1.** Key Modeling Parameters and Inputs for an Example Patient

Parameter	Mean value	Distribution for PSA	Lower	Upper	Source
**Polypill**					
Annual price, $^a^^,^^b^	463	γ	349	577	AHRQ^25^
Mean SBP reduction, mm Hg^b^^,^^c^	19.9	γ	18.5	21.3	Law et al,^26^ 2009; Law et al,^27^ 2003
LDL-C reduction, %^d^	37.1	β	27.8	46.3	Adams et al,^28^ 2015
Annual probability of adverse event					
Any BP medication-related AE	0.0659	β	0.0551	0.0955	Law et al,^26^ 2009; Law et al,^27^ 2003; CDC^29^
Intolerable BP medication-related AE	0.0157	β	0.0046	0.0354	Law et al,^26^ 2009; Law et al,^27^ 2003; CDC^29^
Serious BP medication-related AE	0.0131	β	0.0108	0.0166	Xie et al,^30^ 2016; Wright et al,^31^ 2015
Any statin-related AE	0.0050	β	0.0024	0.0076	Cai et al,^32^ 2021
Intolerable statin-related AE	0.1190	β	0.0893	0.1488	Zhang et al,^33^ 2013
Serious statin-related AE	0.0001	NA	0.00008	0.00014	Newman et al,^34^ 2019
Statin-related diabetes, absolute risk increase	0.0050	NA	0.0010	0.0001	Finegold et al,^35^ 2014
Adherence and discontinuation					
Treatment adherence	0.860	β	0.810	0.910	Muñoz et al,^10^ 2019
Discontinuation 1st year, newly initiated polypill	0.220	Weibull	0.165	0.275	
**Usual care BP-reducing medications**					
Annual price of BP-reducing medication^a^^,^^b^					
1 Half standard dose	117	γ	101	133	AHRQ^25^
⋮^e^	⋮	⋮	⋮	⋮	
4 Standard doses	515	γ	408	621	
Example SBP reduction, mm Hg^b^^,^^c^^,^^d^					
1 Half standard dose	6.7	γ	6.1	7.2	Law et al,^26^ 2009; Law et al,^27^ 2003
⋮	⋮	⋮	⋮	⋮	
4 Standard doses	34.2	γ	19.2	49.1	
RR per 10 mm Hg SBP reduction					
HF, nonischemic	0.72	β	0.67	0.78	Ettehad et al,^36^2016
CHD	0.83	β	0.78	0.88	
Stroke	0.73	β	0.69	0.73	
Annual probability of any adverse event^a^^,^^d^					
1 Half standard dose	0.031	β	0.001	0.093	Law et al,^26^ 2009; Law et al,^27^ 2003; CDC^29^
⋮	⋮	⋮	⋮	⋮	
4 Standard doses	0.147	β	0.133	0.164	
Proportion of adverse events that are intolerable, by No. of classes taken^d^					
1 Class	0.005	β	0.001	0.011	Law et al,^26^ 2009; Law et al,^27^ 2003; CDC^29^
⋮	⋮	⋮	⋮	⋮	
4 Classes	0.023	β	0.007	0.050	
Proportion of adverse events that are serious, by No. of classes taken^d^					
<2 classes	0.009	β	0.006	0.010	Xie et al,^30^ 2016; Wright et al,^31^ 2015
≥2 classes	0.013	β	0.011	0.017	
Adherence^d^					
1 Class	0.900	β	0.681	0.963	Claxton et al,^37^ 2001; Vrijens et al,^38^ 2008
⋮	⋮	⋮	⋮	⋮	
≥4 Classes	0.747	β	0.669	0.881	
Discontinuation					
Discontinuation 1st year, newly initiated BP medication	0.430	Weibull	0.323	0.538	Bellows et al,^19^ 2019; Vrijens et al,^38^ 2008; Lin et al,^39^ 2016
**Usual care intermediate-intensity statin**					
Annual price, $^a^^,^^b^	112	γ	45	179	AHRQ^25^
LDL-C reduction, %^b^	37.1	β	27.8	46.3	Adams et al,^28^ 2015
Statin-induced diabetes, risk increase, %	0.500	Log-normal	0.001	0.010	Finegold et al,^35^ 2014
RR per 1.0 mmol/L (38.67 mg/dL) LDL-C reduction					
CHD	0.76	β	0.73	0.79	Mihaylova et al,^40^ 2012
Stroke	0.85	β	0.80	0.89	
Annual probability of adverse event					
Any	0.0050	β	0.0024	0.0076	Cai et al,^32^ 2021
Intolerable	0.0010	β	0.0893	0.1488	Cai et al,^32^ 2021
Serious	0.0001	NA	0.00008	0.00014	Newman et al,^34^ 2019
Adherence and discontinuation					
Treatment adherence	0.792	β	0.694	1.000	NA^f^
Discontinuation 1st year, newly initiated statin therapy	0.430	Weibull	0.323	0.538	Bellows et al,^19^ 2019; Vrijens et al,^38^ 2008; Lin et al,^39^ 2016
**Physician visits and other parameters**					
Cost, preventive physician visit, $	82	γ	75	105	AHRQ^25^
Preventive physician visit regularity^d^					
Weeks between office visits (BP screening only)	66.8	γ	46.8	86.8	AHRQ^25^
Weeks between office visits (BP and LDL-C screening)	111.3	γ	74.5	147.5	
Cost of adverse event physician visits, $^a^^,^^d^					
Intolerable adverse event	120	γ	110	155	AHRQ^25^
Serious adverse event	162	γ	148	210	
Other parameters					
Annual pill-taking disutility	0.002	β	0.000	0.004	Kohli-Lynch et al,^41^ 2019; Hutchins et al,^42^ 2015; Hutchins et al,^43^ 2015
Discount rate, health and costs, %	3.0	NA	1.0	5.0	US Bureau of Labor^22^

Abbreviations: AE, adverse event; AHRQ, Agency for Healthcare Research and Quality; BP, blood pressure; CDC, US Centers for Disease Control and Prevention; CHD, coronary heart disease; HF, heart failure; LDL-C, low-density lipoprotein cholesterol; NA, not available; RR, relative risk; SBP, systolic blood pressure.

^a^ All costs inflated to US dollars 2023 from baseline year using consumer price index for medical care.^23^

^b^ For patients with full adherence.

^c^ For patients with baseline 150 mm Hg (increases above this value, reduces below).

^d^ Varied concurrently in 1-way sensitivity analysis.

^e^ Symbol indicates that there are steps in medication between these points, which are presented in full in eTables 6 and 7 in Supplement 1.

^f^ Recalibrated to match 1-year SBP and LDL-C reductions for polypill vs usual care.

The model accounted for risk of permanent medication discontinuation and imperfect adherence (ie, proportion of pills taken as prescribed by persistent users).^13,14,15,19,44^ These rates came from published literature,^39^ prior analyses of antihypertensive medication discontinuation,^19,38^ and SCCS Polypill Trial data (eMethods and eFigure 2 in Supplement 1).^45^ Individuals could discontinue treatment for up to 5 years after initiation (eFigure 2 in Supplement 1). Among those remaining on treatment, imperfect adherence attenuated SBP and LDL-C reductions. Adherence to BP medication ranged from 90% (1 medication) to 75% (4 or more medications),^37,38^ and statin adherence was 90%. Polypill adherence was 86%, as observed in the trial (eFigure 2 in Supplement 1).^10^

Blood pressure and LDL-C medication may cause tolerable, intolerable, and serious adverse events. Adverse event rates for BP medications were derived from published literature and NHANES.^13,14,15,19,44^ Adverse events rates for statin therapy were derived from a published meta-analysis, including a slight increased risk of diabetes.^32,35^ Adverse event rates for the polypill were assumed to match those of 1.5 standard dose antihypertensive medications plus statin therapy.

An annual pill-taking disutility of 0.002 QALYs was applied to all individuals taking daily medication.^18,43^ This value likely does not vary with the number of pills taken daily.^42,43^ The price of BP medications and statins were estimated in MEPS 2021 (Table 1 and eTable 7 in Supplement 1).^10,19,22,23,25,26,27,28,29,30,31,32,33,34,35,36,37,38,39,40,41,42,43^ In the base case, we used an annual price of $463 for the polypill. This represented the combined median price of 3 guideline-recommended half-standard dose BP-reducing medications and intermediate-intensity statin therapy, weighted by utilization of comparable medications. Sensitivity analyses considered the cost-effectiveness of polypill treatment across a range of prices. These included using the mean MEPS estimates for the price of polypill constituent medications ($872 per year),^25^ reflecting use of high-price brand name preventive medications, and the national average drug acquisition cost for the polypill ingredients ($222 per year), reflecting the average purchase price paid by retail community pharmacies in the US (eTable 7 in Supplement 1).^46,47^

### Calibration/Validation

The CVDPM was calibrated to reproduce contemporary CVD incidence and total event rates; in addition, the CVDPM was calibrated to reproduce CVD and non-CVD mortality rates for non-Hispanic Black US adults (eMethods and eFigure 3 in Supplement 1). Event rates were calibrated to this population as over 95% of trial participants were Black. Calibration targets were derived from the NHLBI-PCS, Centers for Disease Control and Prevention, National Hospital Discharge Survey, National Inpatient Sample, and National Vital Statistics System.^13,14,15^ We calibrated medication adherence and treatment intensification rates to replicate 1-year BP and LDL-C reductions observed in the trial and the attenuation of risk factor control observed in a meta-analysis of single-pill antihypertensive medication trials (ie, 35% lower BP reduction at 5 vs 1 year) (eTable 5 and eFigure 4 in Supplement 1).^13,14,15^

### Statistical Analysis

This analysis adhered to best-practice recommendations for health economic evaluation studies (eTable 8 in Supplement 1).^48^ Each run of the model simulated 100 000 individuals, a number that produced stable estimates for the primary outcomes (eFigure 5 in Supplement 1). All outcomes were presented using the mean and 95% uncertainty interval (UI) from 100 probabilistic runs of the model. In each run, parameters were sampled from prespecified distributions.

One-way sensitivity analyses (ie, changing model inputs one-by-one while holding all others constant) examined the impact of model inputs on outcomes. In the base case, all individuals in the polypill arm commenced treatment, regardless of baseline medication. In a scenario analysis, only those with baseline medication initiated polypill therapy. The aim of this analysis was to directly compare polypill treatment to separate BP and statin medication, omitting individuals with no treatment in the usual care arm. A second scenario analysis increased pill-taking disutility by 0.001 for patients taking more than one medication.^42^ We also estimated the cost-effectiveness of polypill treatment over a range of time horizons. Data analyses were performed using TreeAge software, version 2024 (TreeAge Inc).

## Results

### Base Case Analysis

The trial-representative cohort included 100 000 individuals (mean [SD] age, 56.9 [5.9] years; 61 807 female [61.8%]; 38 193 male [38.2%]), probabilistically sampled from 3720 NHANES participants. The base-case analysis compared polypill treatment to usual care in a SCCS Polypill Trial–representative cohort of 100 000 individuals over 10 years. Polypill treatment was estimated to prevent 2180 (95% UI, 1900-2510) incident and 2740 (95% UI, 2370-3250) total CVD events (Table 2 and eTable 9 in Supplement 1). One CVD event would be prevented per 36 patients who initiated polypill treatment. Polypill treatment was estimated to reduce primary care visits by 14 900 (95% UI, 13 100-17 000). Serious adverse events were estimated to increase by 881 (95% UI, 826-950); that is, 1 serious adverse event was projected to occur per 114 individuals treated with the polypill. Polypill treatment was projected to produce 1190 (95% UI, 287-2159) discounted QALYs, at a cost of approximately $10 152 000, and 847 (95% UI, −576 to 2341) undiscounted life-years.

**Table 2.** Cost-Effectiveness Outcomes Over 10 Years (100 000 Simulated Individuals)

Outcomes	Polypill trial population (95% UI)		
	Usual care	Polypill	Incremental, polypill vs usual care
Discounted costs, $000s	9 022 295 (8 995 824 to 9 045 413)	9 032 447 (9 011 510 to 9 053 567)	10 152 (−13 330 to 36 618)
Discounted QALYs	601 599 (600 642 to 602 526)	602 786 (602 081 to 603 530)	1187 (287 to 2159)
ICER, $/QALY	NA	NA	8556
Total CVD events	21 964 (21 679 to 22 347)	19 225 (18 959 to 19 477)	−2738 (−3246 to −2374)
Life years	930 415 (929 103 to 931 725)	931 262 (930 205 to 932 403)	847 (−576 to 2341)
Probability polypill preferred strategy at cost-effectiveness threshold of:			
$50 000/QALY	99%		
$100 000/QALY	100%		
$150 000/QALY	100%		

Abbreviations: CVD, cardiovascular disease; ICER, incremental cost-effectiveness ratio; NA, not applicable; QALY, quality-adjusted life-year; UI, uncertainty interval.

**Figure 2.**
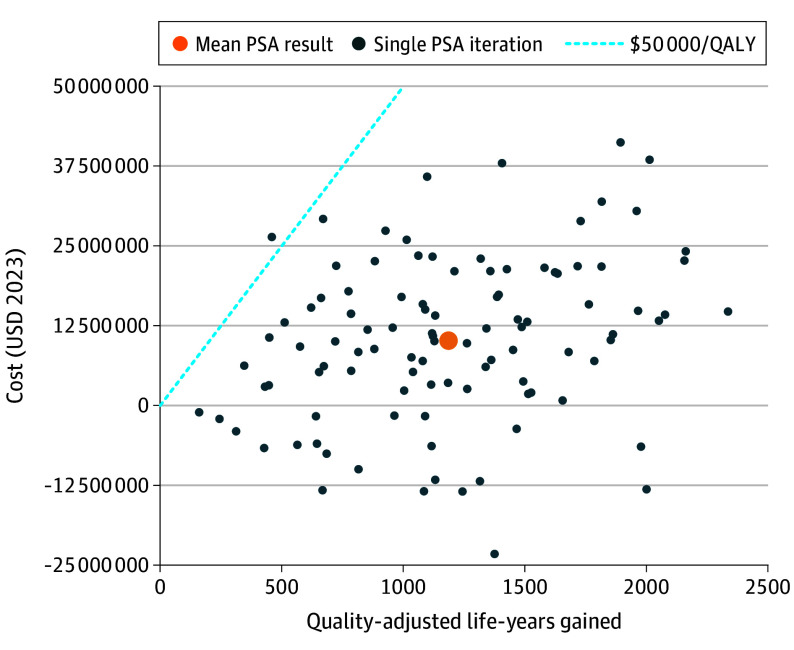
Cost-Effectiveness Scatterplot PSA indicates probabilistic sensitivity analysis; QALY, quality-adjusted life-year; USD, US dollar.

**Figure 3.**
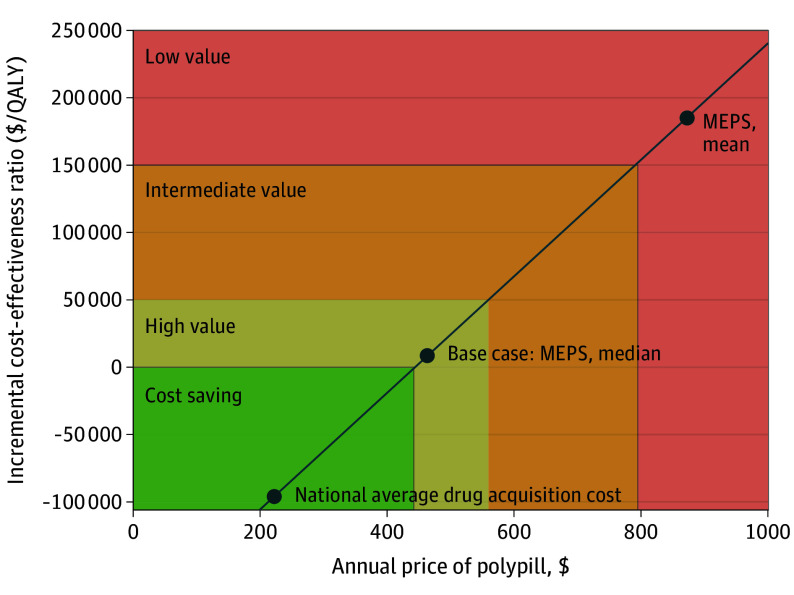
Incremental Cost-Effectiveness Ratio vs Price of Polypill MEPS indicates Medical Expenditure Panel Survey; QALY, quality-adjusted life-year. Colors represent the following: green, high value/cost saving; orange, intermediate value; red, low value.

At the base-case price of $463 per year, polypill treatment was estimated to cost $8560 per QALY gained compared with usual care. It was estimated to be high value (ie, ICER<$50 000 per QALY gained) in 99% of probabilistic model runs (Table 2, Figure 2, and eFigure 6 in Supplement 1). Polypill treatment was projected to increase medication costs by $136 700 000 (95% UI, $136 100 000-$137 500 000). These costs were offset by $61 300 000 (95% UI, $49 700 000-$76 800 000) savings on acute CVD care and $84 900 000 (95% UI, $73 500 000-$99 300 000) savings on chronic CVD care, representing a reduction of around 12% in expenditure on CVD care compared to usual care (eTable 9 in Supplement 1). At the national average drug acquisition cost of polypill ingredients, polypill treatment would be cost saving. This indicates increased QALYs with reduced net costs. At the mean MEPS price, accounting for use of high-cost brand name medications, polypill treatment would have an ICER of $185 000 per QALY gained and be low value (Figure 3).

### Sensitivity and Scenario Analyses

In a secondary analysis, we simulated scaling the cardiovascular polypill up to all 3 602 427 trial-eligible non-Hispanic Black US adults (mean [SD] age, 55.4 [7.6] years; 2 006 597 female [55.7%]; 1 595 830 male [44.3%]), probabilistically sampled from 782 individuals in the NHANES dataset. In almost all sensitivity analyses, polypill treatment was estimated to remain high value (eFigure 7 in Supplement 1). One-way sensitivity analysis indicated that adherence and price had the largest impact on cost-effectiveness of polypill treatment. Polypill treatment would be cost saving at annual prices below $443 (Figure 3) and high value at prices below $559.

When limited to individuals with baseline medication use and when pill-taking disutility was increased for individuals with more than 1 medication in the usual care arm, polypill treatment retained high value (eTable 10 in Supplement 1). The cost-effectiveness of polypill treatment was sensitive to the time horizon, becoming high value compared with usual care approximately 7 years after treatment initiation (eFigure 8 in Supplement 1). Over a lifetime, polypill treatment increased average life expectancy by over 3 months and remained highly cost-effective (eTable 10 in Supplement 1).

### Secondary Analysis of All Polypill-Eligible Non-Hispanic Black US Adults

Compared with the trial-representative cohort, the population of polypill-eligible non-Hispanic Black adults had fewer women, a lower proportion with annual household income of less than $15 000 per year (26.3% vs 67.3%), higher mean LDL-C level (mean [SD], 119.3 [32.0] mg/dL vs 113.5 [29.5] mg/dL), a higher rate of diabetes (17.7% vs 15.0%), less baseline statin (13.6% vs 17.9%) and BP medication (37.0% vs 49.3%) use, lower mean age, and lower 10-year ASCVD risk (mean [SD], 11.0 [9.0] vs 12.5 [9.5]) (eTable 1 in Supplement 1). In the 3.6 million non-Hispanic Black adults meeting the trial eligibility criteria, polypill treatment was estimated to prevent 90 700 (95% UI, 77 800-104 000) CVD events and gain 39 100 (95% UI, 6590-75 800) QALYs over 10 years. It was estimated to cost $13 400 per QALY gained and was high value in 97% of the probabilistic model runs (eTable 11 in Supplement 1). Polypill treatment remained high value at annual prices up to $550 (eFigure 9 in Supplement 1). Although polypill treatment was highly cost-effective in all household income to poverty ratio subgroups, it yielded more QALYs and lower ICERs among individuals with a household income to poverty ratio of 3.0 or less vs greater than 3.0, suggesting it could cost-effectively reduce income-related health inequalities (eTable 12 in Supplement 1). The subgroup with household income to poverty ratio of 1.5 to 3.0 had higher baseline 10-year CVD risk (11.8%) than the subgroup with an income to poverty ratio of less than 1.5 (11.1%), which resulted in more CVD events prevented with polypill treatment over 10 years.

## Discussion

The SCCS Polypill Trial showed that polypill treatment effectively controls CVD risk factors in a population with a majority of Black and low-income individuals. We used a computer simulation model to project health economic outcomes of the trial over 10 years. Fewer physician visits would occur with the polypill, due to a higher number of treated patients with controlled BP. A cardiovascular polypill priced at $463 per year would be high value, and polypill treatment would reduce health care costs when priced at $443 or less per year. Further, we projected that scaling the polypill intervention up to 3.6 million non-Hispanic Black US adults who meet the trial eligibility criteria would maintain its high value.

The polypill would be highly cost-effective among individuals eligible for the SCCS Polypill Trial identified opportunistically. However, it may be challenging for health systems to reach underserved populations. Although such costs were not considered in our analysis, polypill treatment would remain high value if around $490 were spent per individual on patient identification efforts (eg, community outreach, screening programs).

Previous studies indicate that polypills are cost-effective in the secondary prevention of CVD, with some evidence of cost-effectiveness in primary prevention.^49,50^ The SBP and LDL-C reductions observed in the SCCS Polypill Trial were similar in scale to those seen in different settings, suggesting that our finding may be applicable to a wider population.^11^ The SCCS Polypill Trial was unique in evaluating the polypill in a predominantly low-income and Black population without established CVD and who receive health care at a federally qualified community health center.^10^ This population may have lower preventive medication adherence than the general population and, therefore, may achieve particular benefit from the polypill treatment paradigm.^51^ However, the polypill would likely be cost-effective in other underserved populations, including other racial or ethnic groups. Our results may be particularly relevant to other patient groups with low rates of treatment persistence and adherence, including community-dwelling older adult patients with chronic polypharmacy^52^ and younger adults with elevated cardiovascular risk factors.^53^

We projected that polypill treatment would increase medication, adverse event, and non-CVD–related health care costs. Approximately 93% of these costs would be recovered after 10 years through reduced CVD care and physician office visit costs. The polypill consists of off-patent, generically available molecules whose active pharmaceutical ingredients are manufactured by multiple companies. Our findings suggest that a polypill priced at less than $1.50 per day could be profitable and sustainable. Despite evidence supporting the cost-effectiveness of polypills, no manufacturer has invested the millions of dollars required to gain US Food and Drug Administration approval. This study may be part of the process of signaling demand for the cardiovascular polypill to pharmaceutical manufacturers.

The Inflation Reduction Act (2022) allows Medicare to directly negotiate prices for a select group of single-source brand name medications.^54^ As the polypill would not be eligible to be included in price negotiations, our analysis demonstrates the value of expanding Medicare’s negotiation remit. Additionally, the value of fulfilling the health equity goal of reduced disparities in CVD should be considered alongside standard measures of cost-effectiveness.^55^

### Limitations

Our cost-effectiveness results were highly robust in sensitivity analyses. However, we adopted a health care sector perspective, only including costs related to health care goods and services in our analysis.^22^ Preventing CVD events will likely produce further economic benefits, including reduced societal costs associated with absenteeism and presenteeism. Future analyses may wish to incorporate societal costs, although it is unclear if health care payers should pay for nonhealth benefits.^56^ We also limited the time horizon of our analysis to 10 years. After this time, treatment intensification would be required for a substantial proportion of polypill users. Intensification could be achieved through the addition of medications to the polypill or by producing more intensive polypill formulations. Although evidence for such a treatment does not exist, it may extend the duration of polypill benefit beyond the time horizon of our analysis.

In the primary analysis, we weighted the simulated population to represent the SCCS Polypill Trial population on demographic, including household income, and clinical characteristics. However, NHANES does not include data describing whether respondents primarily receive care in community health centers, which represents underserved individuals and is where individuals were identified for the trial. Therefore, our analysis of non-Hispanic Black adults who meet the trial eligibility criteria may not be representative of this underserved population nationally. This may be evidenced by the lower proportion of individuals with annual household income of less than $15 000 per year in that population.

## Conclusions

The SCCS Polypill Trial showed that cardiovascular polypills are effective at controlling CVD risk factors in a low-income, majority Black population with limited access to preventive health care services. In this economic evaluation, using a computer simulation model, we projected that the polypill would be high value in this population if priced based on its component medications ($463 per year) and may reduce income-related health disparities.

## Notes

Supplement 1.
eMethods.
**eTable 1.** Baseline Characteristics of Polypill Trial and the Simulated Cohorts **eTable 2.** Physician Office Visit Frequency and Blood Pressure Measurement Error **eTable 3.** Probability of Incident CVD and Non-CVD Mortality **eTable 4.** Health-Related Costs and Utility Values in CVDPM **eTable 5.** Validation of 1-Year Blood Pressure and Lipid Outcomes vs Polypill Trial **eTable 6.** Modeled SBP and LDL-C Reductions, Risk Adjustments, and Adverse Events **eTable 7.** Medication Prices **eTable 8.** Consolidated Health Economics Evaluation Reporting Standards Checklist **eTable 9.** Disaggregated Economic and Clinical Outcomes, Polypill Population **eTable 10.** Cost-Effectiveness in Scenario Analyses **eTable 11.** Cost-Effectiveness: Trial-Eligible Non-Hispanic Black Population **eTable 12.** Extended Cost-Effectiveness Results **eFigure 1.** Inclusion and Exclusion Criteria for NHANES Population **eFigure 2.** Discontinuation Curve, Polypill vs Usual Care (Both BP and LDL-C Medications) **eFigure 3.** Recalibration/Validation Figures, Women and Men: Death Rates, Total Event Rates, and Incident Event Rates **eFigure 4.** Calibrating Treatment Effect **eFigure 5.** Cost-Effectiveness Outcomes With Different Numbers of Simulated Individuals **eFigure 6.** Cost-Effectiveness Acceptability Curve (Polypill Trial Population) **eFigure 7.** Tornado Diagram **eFigure 8.** ICER: Polypill vs Usual Care at Range of Time Horizons **eFigure 9.** Polypill Price vs ICER: Trial-Eligible Non-Hispanic Black Individuals
eReferences.

Supplement 2.
Data Sharing Statement.
