# Supplementary material for: Cost-Effectiveness of a Polypill for Cardiovascular Disease Prevention in an Underserved Population
Source: JAMA Cardiol. 2025 Jan 8;10(3):224–33. doi: 10.1001/jamacardio.2024.4812 (PMC11904704; doi:10.1001/jamacardio.2024.4812)
Supplement: Supplement 1. — eMethods. eTable 1. Baseline Characteristics of Polypill Trial and the Simulated Cohorts eTable 2. Physician Office Visit Frequency and Blood Pressure Measurement Error eTable 3. Probability of Incident CVD and Non-CVD Mortality eTable 4. Health-Related Costs and Utility Values in CVDPM eTable 5. Validation of 1-Year Blood Pressure and Lipid Outcomes vs Polypill Trial eTable 6. Modeled SBP and LDL-C Reductions, Risk Adjustments, and Adverse Events eTable 7. Medication Prices eTable 8. Consolidated Health Economics Evaluation Reporting Standards Checklist eTable 9. Disaggregated Economic and Clinical Outcomes, Polypill Population eTable 10. Cost-Effectiveness in Scenario Analyses eTable 11. Cost-Effectiveness: Trial-Eligible Non-Hispanic Black Population eTable 12. Extended Cost-Effectiveness Results eFigure 1. Inclusion and Exclusion Criteria for NHANES Population eFigure 2. Discontinuation Curve, Polypill vs Usual Care (Both BP and LDL-C Medications) eFigure 3. Recalibration/Validation Figures, Women and Men: Death Rates, Total Event Rates, and Incident Event Rates eFigure 4. Calibrating Treatment Effect eFigure 5. Cost-Effectiveness Outcomes With Different Numbers of Simulated Individuals eFigure 6. Cost-Effectiveness Acceptability Curve (Polypill Trial Population) eFigure 7. Tornado Diagram eFigure 8. ICER: Polypill vs Usual Care at Range of Time Horizons eFigure 9. Polypill Price vs ICER: Trial-Eligible Non-Hispanic Black Individuals eReferences. [file jamacardiol-e244812-s001.pdf]

Supplementary Online Content

Kohli-Lynch CN, Moran AE, Kazi DS, et al. Cost-effectiveness of a polypill for cardiovascular disease prevention in an underserved population. *JAMA Cardiol*. Published online January 8, 2025. doi:10.1001/jamacardio.2024.4812

eMethods.

eTable 1. Baseline Characteristics of Polypill Trial and the Simulated Cohorts

eTable 2. Physician Office Visit Frequency and Blood Pressure Measurement Error

eTable 3. Probability of Incident CVD and Non-CVD Mortality

eTable 4. Health-Related Costs and Utility Values in CVDPM

eTable 5. Validation of 1-Year Blood Pressure and Lipid Outcomes vs Polypill Trial

eTable 6. Modeled SBP and LDL-C Reductions, Risk Adjustments, and Adverse Events

eTable 7. Medication Prices

eTable 8. Consolidated Health Economics Evaluation Reporting Standards Checklist

eTable 9. Disaggregated Economic and Clinical Outcomes, Polypill Population

eTable 10. Cost-Effectiveness in Scenario Analyses

eTable 11. Cost-Effectiveness: Trial-Eligible Non-Hispanic Black Population

eTable 12. Extended Cost-Effectiveness Results

eFigure 1. Inclusion and Exclusion Criteria for NHANES Population

eFigure 2. Discontinuation Curve, Polypill vs Usual Care (Both BP and LDL-C Medications)

eFigure 3. Recalibration/Validation Figures, Women and Men: Death Rates, Total Event Rates, and Incident Event Rates

eFigure 4. Calibrating Treatment Effect

eFigure 5. Cost-Effectiveness Outcomes With Different Numbers of Simulated Individuals

eFigure 6. Cost-Effectiveness Acceptability Curve (Polypill Trial Population)

eFigure 7. Tornado Diagram

eFigure 8. ICER: Polypill vs Usual Care at Range of Time Horizons

eFigure 9. Polypill Price vs ICER: Trial-Eligible Non-Hispanic Black Individuals

eReferences.
This supplementary material has been provided by the authors to give readers additional information about their work.

© 2025 Kohli-Lynch CN et al. *JAMA Cardiology*.

Supplemental Methods

Blood Pressure Control-Cardiovascular Disease Policy Model

A discrete event simulation version of the Cardiovascular Disease (CVD) Policy Model (CVDPM), which simulates the healthcare delivery and patient interactions with healthcare providers in the U.S., was used. The discrete event simulation version of the CVDPM has been used in previous cost-effectiveness analyses of preventive interventions for CVD.[1–3] The CVDPM simulates time continuously, progressing from one event to the next. The events included are: (i) physician office visits (including polypill trial protocol visits in first year and all other physician office visits where BP and cholesterol could be managed), (ii) antihypertensive and statin medication-related adverse events, (iii) antihypertensive and statin medication discontinuation for any reason, (iv) fatal or non-fatal CVD events, and (v) non-CVD death. When an event occurs, the model stores healthcare costs and quality of life outcomes, updates patient characteristics, and determines the time to the next event. In the absence of any event within a given year, the model updates patient characteristics, CVD risk factors, and predicts time to a CVD event and non-CVD death given these changes.

Competing risks Cox proportional hazards functions derived from the National Heart, Lung, and Blood Institute Pooled Cohorts Study (NHLBI-PCS) are used to predict incident CVD events and non-CVD mortality.[1,4,5] The model was calibrated to match CVD event and mortality rates of non-Hispanic Black adults in the U.S. from the NHLBI-PCS, Centers for Disease Control and Prevention, National Hospital Discharge Survey, National Inpatient Sample, and National Vital Statistics System (Figure S3), and is cross validated against the original, dynamic population version of the CVDPM.[2,6,7]

Simulation Cohort and CVD Risk Factor Trajectories
The model is populated by a cohort of individuals from the 1999-2018 National Health and Nutrition Examination Survey (NHANES) that are probabilistically selected, with replacement, to be included in analyses. NHANES is a large-scale, cross-sectional nationwide survey of health and nutritional status in which individuals are selected for inclusion using a multistage probability sampling design. The probability sampling design allowed for oversampling of low-response demographics. The weighted NHANES-based estimates reflect the civilian, non-institutionalized U.S. population. We used survey, examination, and laboratory data for key CVD risk factors from NHANES respondents aged 40-75 years to match the SCCS Polypill Trial.

From NHANES, we included individuals with complete data for the following CVD risk factors: age, systolic blood pressure (SBP), diastolic blood pressure (DBP), current antihypertensive medication use (yes/no), low-density lipoprotein cholesterol (LDL-C), high-desnity lipoprotein cholesterol (HDL-C), total cholesterol, current lipid-lowering medication use (yes/no), tobacco smoking (cigarettes per day), BMI, serum glucose, diabetes status (yes/no) and serum creatinine.

Fitting risk factor exposure trajectories from age 20 years until age 89 years or death
We imputed lifetime trajectories (each year from age 18 to 99 years) for each of the CVD risk factors described above in individuals in the NHLBI Pooled Cohort Project. The details of this approach are described elsewhere.[4,8] Briefly, we leveraged the risk factor patterns observed in the younger cohorts to impute unobserved early adult exposures in the older cohorts and vice versa. We used a series of linear

© 2025 Kohli-Lynch CN et al. *JAMA Cardiology.*

mixed models to estimate latent trajectories underlying the observed values for each participant, and imputed risk factor levels annually from age 18 years until age 99 or death for each participant.

Reweighting NHANES for Analyses
Probabilistic sample weights are available for all NHANES individuals that provided a fasting blood sample. These weights were assigned to enable probability-weighted analyses which are representative of the non-incarcerated U.S. adult population.

In our base case analysis, we aimed to simulate polypill treatment in a cohort comparable to the SCCS polypill trial population. To do this, we redefined probability weights for individuals in the NHANES cohort. This was a two-step process, completed using *R* software (Version 4.2.2; Vienna, Austria). We excluded individuals who did not meet the trial inclusion criteria: individuals aged 40-75 years, SBP ≥120 mm Hg, LDL-C <190 mg/dL, eGFR ≥60 mL/min/1.73 m^2, potassium <5.5 mmol/L, not pregnant, current use of ≤2 BP-lowering medications, and no reported history of CVD, cancer, liver disease, or insulin-dependent diabetes.

Next, we reweighted remaining NHANES individuals to create a cohort with covariate mean and SD values similar to those reported for the polypill trial (Table S1). We used a propensity score-based approached, employing the *ps()* function from the *twang* package in *R*.[9] We aimed to replicate mean and SD values from the polypill trial and assumed the shape of probability distributions for key covariates. The covariates employed in this process (with corresponding probability distributions in parentheses) were: age (gamma, truncated between 45 and 75), SBP (gamma, truncated below 120 mm Hg), LDL-C (gamma, truncated above 190 mg/dL), race, sex, diabetes smoker – current, antihypertensive medication use, statin use, and income <$15,000/year (binomial), BMI, DBP, and HDL-C (gamma), and ASCVD risk (beta). The propensity score method weights were then recalibrated to produce a probability-weighted cohort of NHANES participants with baseline characteristics representative of those who participated in the polypill trial population.

In a secondary analysis, we assessed the cost-effectiveness of polypill treatment in a population of non-Hispanic Black US adults meeting the polypill trial eligibility criteria. For this analysis, we only included non-Hispanic Black NHANES participants and used the NHANES survey weights to calculate the sampling probabilities.

Risk of CVD Events and Non-CVD Death
Individuals in the model are at risk of multiple CVD events: coronary heart disease – including myocardial infarction (MI), other coronary heart disease (including non-MI acute coronary syndrome, unstable angina, and stable angina), and cardiac arrest; new-onset ischemic or non-ischemic heart failure (HF); and stroke. The risk of incident coronary heart disease (CHD), non-ischemic HF, or stroke event and for non-CVD death were derived from competing risks Cox proportional hazards functions in the NHLBI-PCS (Table S3).[2,5–7] The Cox proportional hazards models account for age, sex, race, SBP, DBP, body mass index, low-density lipoprotein cholesterol (LDL-C), high-density lipoprotein cholesterol (HDL-C), smoking status, estimated glomerular filtration rate (eGFR), and diabetes status. Each patient’s detreated SBP and LDL-C (i.e., values if they never received treatment at any time) and observed values for other CVD risk factors were used to estimate underlying risk of CVD.

Risk of recurrent and secondary CVD events, revascularization procedures, and case fatality rates were derived from the Centers for Disease Control and Prevention, National Hospital Discharge Survey,

© 2025 Kohli-Lynch CN et al. *JAMA Cardiology.*

National Inpatient Sample, and National Vital Statistics System, and the original dynamic population version of the CVDPM.

Reductions in SBP and LDL-C when medications are initiated or intensified are associated with reductions in the risk of CVD events. The risk reductions from published meta-analysis per 10-mmHg SBP reduction and 38.67 mg/dL (1.0 mmol/L) LDL-C reduction from the detreated SBP and LDL-C, respectively are applied to the underlying risk of CVD.[10,11]

Risk of Medication-Related Adverse Events

The probability of any adverse events (AEs), intolerable AEs (i.e., requiring antihypertensive discontinuation), and serious AEs (i.e., requiring an emergency department visit or hospitalization) with antihypertensive medication, and the case fatality rate associated with antihypertensive serious AEs, are described elsewhere.[2] The risk of AEs is associated with the number of full- and half-standard dose antihypertensive medications in an individual's regimen were estimated from published meta-analyses, other published literature, and NHANES.[12–15]

Risk of adverse events for statin therapy were defined in the same way as for BP-reducing medication (i.e., any, intolerable, and serious adverse events). The probability of these events were derived from a published meta-analysis (Table S6).[16] Individuals receiving LDL-C medication also experienced a 0.5% absolute increase in diabetes risk.[17] Patients with statin-related diabetes experienced increased costs and increased risk of CVD events, both largely offset by the preventive benefit of statins.[17]

Processes of Care

Differences in the polypill intervention and usual care arms are accounted for in the CVDPM by differences in prescribed medications and processes of care. In the usual care arm, individuals continue their baseline medication and may attend physician visits and, when eligible, receive additional medications, as described below. In the polypill intervention arm, all individuals commence a polypill at baseline and attend screening visits at the same regularity as those in usual care after treatment initiation. In both the polypill and usual care arms, individuals undergo 'study visits' at 6 weeks and 1-year, to replicate the screening protocol of the polypill trial.

Physician Visit Frequency

For both treatment arms, the time between usual care physician visits was derived from the mean number of hypertension-related office visits per year from the Medical Expenditure Panel Survey (MEPS) available from the AHRQ. As in prior analyses, the time between physician visits with an uncontrolled BP was modified to account for differences due to individual and visit characteristics (e.g., increased SBP and DBP since last visit, age, and treatment intensification all reduced the number of weeks until the next visit).[3,18] We disaggregated physician visits into those at which BP was screened and those at which both BP and LDL-C were screened (Table S2).

Risk Factor Measurement Accuracy

The accuracy of BP measurements is captured in the CVDPM by randomly sampling measurement error (i.e., difference between the measured BP and the patient's underlying BP) at each office visit and is described elsewhere.[3,18–20] Measurement error was derived from published literature and is inversely related to the number of office visits a patient has had. We assumed that LDL-C screening was not prone to systematic measurement error.

Probability of Treatment Intensification

As in prior analyses, the probability of antihypertensive treatment intensification after an uncontrolled BP measurement at a physician office visit was derived from published literature and stratified by prior treatment intensification during the simulation and how much the measured BP was above target (Table S2).[3,18] Probability of commencing statin therapy when eligible equaled the proportion of patients in the simulation cohort who were eligible for statin therapy and receiving the treatment at baseline.

Adherence

The CVDPM simulates patient adherence through both the likelihood of discontinuing antihypertensive medications within one year of initiation and imperfect pill-taking adherence (i.e., not taking medications exactly as prescribed).[3,18] In each run of the model. individuals were randomly assigned at baseline the percentage of doses they would take exactly as prescribed for one through five antihypertensive medications.[18] Medication discontinuation rates were derived from published literature of statin usage,[21] prior analyses of antihypertensive medication discontinuation,[3,22] and data from the SCCS polypill trial (Figure S2).[23] Imperfect medication adherence attenuated SBP and LDL-C reductions from treatment. Adherence to the polypill was assumed to be 86%, as observed in the trial.[24] Antihypertensive pill-taking execution reduced as the number of prescribed medications increased, ranging from 90% (one medication prescribed) to 75% (four or more medications).[22,25] Pill-taking execution with statin therapy was assumed to be the same as with one antihypertensive medication, 90%.[22,25] A recalibration factor was applied to adherence in the polypill trial arm to replicate observed 1-year BP and LDL-C reductions (Model Calibration). Pill-taking execution with statin therapy was assumed to be the same as with one antihypertensive medication, 90%.[22,25]

Expected BP and LDL-C Reduction

As in prior analyses, usual care management of BP was assumed to be a traditional “start low and go slow” approach, with initial doses and titration of regimen dependent on BP and individual and visit characteristics.[2,3,6,7,26] BP medication was started as a half-standard dose unless baseline SBP was ≥20 mmHg above goal, then it was started at a standard dose. BP medications were intensified to a standard dose before initiation of additional BP medications.

As in prior analyses, the results of meta-analyses were to estimate the reduction in BP with each full- and half-standard dose medication added to a patient’s regimen.[3,13,18,20,27] As antihypertensive medication discontinuation was accounted for separately from BP reduction in the model, we increased the BP reduction from medications reported in the meta-analysis, to account for the fact that 25% of individuals were reported to discontinue treatment. This was achieved by dividing the expected change in BP by 0.75.[27] The expected BP reduction with incomplete pill-taking execution was derived from an analysis that estimated the percent of the total potential BP reduction achieved by incomplete execution values.[18,28] The potential BP reduction was further divided by the expected percent of BP reduction achieved for each number of antihypertensive medications used to calculate the total potential BP reduction with treatment.[18] If a patient discontinued their antihypertensive medication, it was assumed that they reverted back to their detreated BP. Percentage reduction in untreated LDL-C from intermediate-intensity statin therapy with full adherence was 37%, derived from a meta-analysis of randomized controlled trials of atorvastatin (Table S6).[29]

<u>Cost and Health-Related Quality of Life Inputs</u>

Background Health Care Costs

© 2025 Kohli-Lynch CN et al. *JAMA Cardiology.*

All costs were inflated to 2023 US dollars when needed using the medical care component of the Consumer Price Index for medical care, available from the U.S. Bureau of Labor Statistics.[30] As in previous analyses, annual background healthcare costs of US adults (age ≥18 years), were estimated accounting for differences due to age, sex, race, selected comorbidities, history of CVD events, and long-term care.[2,6,7] Background healthcare costs were defined as total healthcare costs to all payers excluding the cost of CVD events (i.e., emergency room visits and inpatient stays) and treating BP (i.e., antihypertensive medications and BP-related office visits), as they are simulated independently. Pooled 2006-2015 MEPS data available from the AHRQ, the 2010 U.S. Census, 2013-2014 long-term care service utilization from the National Center for Health Statistics, published annual long-term care costs from Genworth Financial Inc., and the 2018 U.S. Renal Data System Annual Report were used to estimate these costs.[31–33]

Medication Costs

The mean cost per unit of oral (i.e., tablet or capsule) antihypertensive and statin medications was estimated using MEPS data available from the AHRQ, which are nationally representative of all U.S. payers (e.g., Medicare, Medicaid, private, Veterans Affairs, patients). MEPS costs include ingredient costs, dispensing fees, taxes, and out-of-pocket costs, but do not include manufacturer rebates. In a sensitivity analysis, we employed national average drug acquisition costs (NADAC) for the polypill ingredients, representing the cap on prices that Medicaid pays for multiple source drug products.[34,35] NADAC represents the utilization-weighted average acquisition cost for medications reimbursed by Medicaid. Drug costs obtained from NADAC represent ingredient costs and do not include dispensing fees, out-of-pocket costs, or manufacturer rebates. Hence, we added a $10.50 dispensing fee per 90-day refill in this analysis, which is the median Medicaid prescribing fee.[36]

CVD Events and Serious Adverse Event Hospitalization Costs

The hospitalization facility costs associated with CVD events and antihypertensive medication-related serious AEs were estimated using National Inpatient Sample (NIS) data from the first quarter of 2012 through the third quarter of 2015 (Table S4).[37] The NIS is available from the AHRQ and provides nationally representative utilization and charge estimates for all payer types. Charges in NIS do not include rehabilitation or long-term acute care hospitals. Charges were converted to costs using the hospital cost-to-charge ratios provided by NIS for each hospital in the sample.

As in Arth et al.,[38] a published professional fee ratio was used, which was developed for use with data from publicly-available discharge datasets such as NIS, to incorporate professional fees and estimate total hospitalization costs.[38,39] Hospitalization costs were converted to 30-day costs using ratios of costs for admissions estimated from the California Office of Statewide Health Planning and Development (OSHPD) to costs over 30-days following discharge measured in the MEPS in 1999-2008.

Health-Related Quality of Life

Health benefits in the CVDPM are accumulated as individuals pass through health states and experience health-related quality of life values assigned to health states (Table S4). Quality-adjusted life years (QALYs) are used to reflect health-related quality of life in the model. This measure reflects the quality and longevity of health, where 1.0 represents perfect health, and QALYs less than 1.0 represent health loss due to illness or imperfect health.

Health-related quality of life inputs were derived from a combination of data on CVD event rates in the U.S.[40,41] and utility weights derived from international analysis.[42] Each health state has an attributed

annual QALY penalty. Additionally, all acute events in the model (e.g., hospitalizations, fatalities) have an associated acute (30-day) QALY penalty. While receiving a treatment, individuals may experience treatment-related disutility.

Model Calibration and Validation

Model CVD Event and Non-CVD Death Recalibration

The CVDPM was recalibrated to reproduce contemporary CVD incidence and total event rates, and CVD and non-CVD mortality rates for, and using a population representative of, non-Hispanic Black adults in the U.S. (Figure S3). The event rate calibration was restricted non-Hispanic Black U.S. adults as more than 95% of SCCS polypill trial population was Black. As in prior analyses, the calibration targets were derived from the NHLBI-PCS, Centers for Disease Control and Prevention, National Hospital Discharge Survey, National Inpatient Sample, and National Vital Statistics System, and cross-validated against the original, dynamic population version of the CVDPM.[2,6,7]

SCCS *Polypill Trial Patient Characteristics and Processes of Care Validation*

To ensure that the CVDPM accurately reproduced the polypill population, baseline characteristics of the simulated population were compared to published polypill trial data (Figure S4, Table S1). We also recalibrated medication adherence and probability of treatment intensification to (i) replicate one-year BP and LDL-C reductions observed in the polypill and usual care arms of the polypill trial and (ii) replicate the attenuation of risk factor control observed in a meta-analysis of fixed-dose BP medication trials (i.e., 35% lower BP reduction at five versus one year; Table S6, Figure S4).[43]

© 2025 Kohli-Lynch CN et al. *JAMA Cardiology.*

Supplemental Tables

eTable 1. Baseline characteristics of polypill trial and the simulated cohorts

Characteristics	Polypill Trial Population	NHANES: Polypill Population (SD[b])	NHANES: Polypill-Eligible N-HB Population[a] (SD[b])
N	303	3,720	782
N represented in population	n/a	33,131,379	3,602,427
Age at Observation (Years)	56.0 ±6.0	56.9 (5.9)	55.4 (7.6)
Male (%)	39.9	38.2	44.3
African American (%)	96.0	97.3	100.0
Systolic Blood Pressure (mmHg)	140 ±17.5	140.2 (15.0)	139.5 (16.4)
Diastolic Blood Pressure (mmHg)	83.0 ±.8.0	82.1 (8.7)	77.3 (11.6)
LDL-C (mg/dL)	113 ±34.6	113.5 (29.5)	119.3 (32.0)
HDL-C (mg/dL)	62.5 ±22.0	62.4 (21.1)	59.4 (17.3)
Body Mass Index (kg/m^2)	30.8 ±8.4	30.7 (7.7)	30.7 (7.4)
Hypertension Medication (%)	53.5	49.3	37.0
Statin at Baseline (%)	17.5	17.9	13.6
Diabetes Mellitus (%)	12.5	15.0	17.7
Current Smoker (%)	48.1	43.0	26.6
Annual Income <$15,000 (%)	74.6	67.3	26.3
Annual Income $15,000-$25,000 (%)	10.9	14.1	10.2
10-Year ASCVD Risk Score	12.7 ±9.5	12.5 (9.5)	11.0 (9.0)

BMI – Body Mass Index, HDL-C – High-Density Lipoprotein Cholesterol, LDL-C – Low-Density Lipoprotein Cholesterol, NHANES – National Health and Nutrition Examination Survey, N-HB – Non-Hispanic Black, SD – Standard Deviation, UI – uncertainty interval

[a]Wider population only includes individuals who meet polypill trial inclusion and exclusion criteria, but was not propensity score matched to polypill trial
[b]Mean PSA iteration-level standard deviation

© 2025 Kohli-Lynch CN et al. *JAMA Cardiology.*

eTable 2. Physician office visit frequency and blood pressure measurement error

Model Input	Mean	SD	Min	Max	Distribution	Source
Office visit frequency						
Off-treatment						
Weeks between BP+Lipid screening	111	36.5	74.5	147.5	Gamma	MEPS[31]
Weeks between BP screening alone	66.8	20.0	46.8	86.8	Gamma	MEPS[31]
On treatment						
Weeks between visits when BP controlled	66.8	20.0	46.8	86.8	Gamma	MEPS[31]
Change in weeks between visits when measured BP uncontrolled						
Age (per year)	-0.147	0.012	-0.117	-0.164	Normal	Bellows et al.[3]
Change in SBP since last visit (per mmHg increase)	-0.052	0.009	-0.074	-0.039	Normal	
Change in DBP since last visit (per mmHg increase)	-0.056	0.008	-0.065	-0.035	Normal	
Antihypertensive medication added at the visit	-2.080	0.199	-2.470	-1.690	Normal	
Intensification probabilities						
Adding/titrating first antihypertensive medication						
SBP ≥160 mmHg or BP >14/90 mmHg with diabetes of chronic kidney disease	0.333	0.032	0.313	0.440	Beta	Bellows et al.[3]
SBP uncontrolled but <160 mmHg or BP uncontrolled but <140/90 mmHg with diabetes or chronic kidney disease	0.208	0.026	0.207	0.310	Beta	
Adding/titrating additional antihypertensive medications	0.130	0.033	0.065	0.195	Beta	
BP measurement accuracy						
Difference between measured and "true" BP by total number of visits and measurements per visit[a]						
SBP						
1 visit with 1 measurement	0.000	8.100	-	-	Normal	Kronish et al.[19] Bryant et al.[2]
1 visit with ≥3 measurements	0.000	6.200	-	-	Normal	

© 2025 Kohli-Lynch CN et al. *JAMA Cardiology.*

Model Input	Mean	SD	Min	Max	Distribution	Source
2 visits with 1 measurement	0.000	5.940	-	-	Normal	
2 visits with ≥3 measurements	0.000	4.390	-	-	Normal	
≥3 visits with 1 measurement	0.000	5.000	-	-	Normal	
≥3 visits with ≥3 measurements	0.000	3.650	-	-	Normal	
DBP						
1 visit with 1 measurement	0.000	5.450	-	-	Normal	Kronish et al.[19] Bryant et al.[2]
1 visit with ≥3 measurements	0.000	4.370	-	-	Normal	
2 visits with 1 measurement	0.000	3.900	-	-	Normal	
2 visits with ≥3 measurements	0.000	3.120	-	-	Normal	
≥3 visits with 1 measurement	0.000	3.180	-	-	Normal	
≥3 visits with ≥3 measurements	0.000	2.550	-	-	Normal	

BP – Blood Pressure, DBP – Diastolic Blood Pressure, SD – Standard Deviation

[a]Increasing the number of visits and measurements per visit increases the diagnostic accuracy of measured BP. At each visit, the difference between the patient's underlying "true" BP and the BP measured at the visit is estimated by sampling from normal distributions with a mean of 0 (i.e., no difference between "true" and measured BP) and a standard deviation that decreases with more visits and more measurements per visit.

© 2025 Kohli-Lynch CN et al. *JAMA Cardiology*.

eTable 3. Probability of incident CVD and non-CVD mortality

Model Input	Mean	Standard Deviation	Distribution	Source
Risk of primary CHD event (logistic risk function)				
Age	0.10178	0.0084	Multivariate Normal	CU-NHLBI PCS: Oelsner et al.[5] Zhang et al.[4] Bryant et al.[2]
Non-Hispanic Black	-0.1979	0.0386		
Former smoker	0.1841	0.0324		
Current smoker	0.4602	0.0639		
Cigarettes per day	0.0064	0.0026		
SBP	0.0130	0.0007		
DM	0.5963	0.0374		
HDL-C	-0.0158	0.0013		
LDL-C	0.0058	0.0004		
eGFR	-0.0058	0.0009		
Current smoker * age	-0.0139	0.0026		
SBP * age	-0.0003	0.00004		
DM * age	-0.0096	0.0026		
HDL-C * age	0.0003	0.00008		
LDL-C * age	-0.0002	0.00003		
Risk of non-ischemic HF (logistic risk function)				
Age	0.1306	0.01066	Multivariate Normal	CU-NHLBI PCS: Oelsner et al.[5] Zhang et al.[4]
Non-Hispanic Black	0.5217	0.0500		
BMI	0.0535	0.0034		
Former smoker	0.2938	0.0433		
Current smoker	0.7945	0.0806		
Cigarettes per day	0.0152	0.0034		
SBP	0.0126	0.0011		
DM	0.6309	0.0479		
LDL-C	-0.0031	0.0006		
eGFR	-0.0114	0.0012		
Non-Hispanic Black * age	-0.0274	0.0040		
SBP * age	-0.0002	0.0001		
DM * age	-0.0139	0.0037		
Risk of primary stroke (logistic risk function)				
Age	0.1420	0.0107	Multivariate Normal	CU-NHLBI PCS: Oelsner et al.[5] Zhang et al.[4]
Non-Hispanic Black	0.4790	0.0611		
Current smoker	0.6011	0.0607		
SBP	0.0204	0.0011		
DM	0.6499	0.0573		
HDL-C	-0.0050	0.0016		
LDL-C	0.0018	0.0007		
eGFR	-0.0044	0.0015		
Non-Hispanic Black * age	-0.0226	0.0044		
Current Smoker * age	-0.0126	0.0042		
SBP * age	-0.0005	0.0001		

© 2025 Kohli-Lynch CN et al. *JAMA Cardiology*.

Model Input	Mean	Standard Deviation	Distribution	Source
Risk of non-CVD death (logistic risk function)				
Age	0.0985	0.0035	Multivariate Normal	CU-NHLBI PCS: Oelsner et al.[5] Zhang et al.[4]
Non-Hispanic Black	0.3496	0.0378		
BMI	-0.0984	0.0121		
BMI2	0.0012	0.0002		
Former smoker	0.2322	0.0310		
Current smoker	0.6883	0.0570		
Cigarettes per day	0.0189	0.0023		
DM	0.3662	0.0397		
eGFR	-0.0047	0.0001		
Non-Hispanic Black * age	-0.0121	0.0025		
BMI2 * age	0.00001	0.00001		
Heart Failure				
Probability, Concurrent HF with MI, by age				
<55	0.1209	n/a	n/a	McManus et al.[44] Spencer et al.[45]
55-64	0.1999	n/a	n/a	
65-74	0.2904	n/a	n/a	
75-84	0.3874	n/a	n/a	
>85	0.4768	n/a	n/a	
Probability, HF within 1 year of MI	0.1613	0.0003	Beta	Hernandez et al.[46] Steg et al.[47]
Annual probability, HF from 1-5 years after MI	0.0861	n/a	n/a	Gerber et al.[48]
Probability, Develop ischaemic HF within five years				
Male				
45-64	0.130	n/a	n/a	Benjamin et al.[49] Hernandez et al.[46] McManus et al.[44] Spencer et al.[45] Steg et al.[47]
65-74	0.200	n/a	n/a	
75+	0.230	n/a	n/a	
Female				
45-64	0.250	n/a	n/a	Benjamin et al.[49] Hernandez et al.[46] McManus et al.[44] Spencer et al.[45] Steg et al.[47]
65-74	0.320	n/a	n/a	
75+	0.190	n/a	n/a	
Concurrent MI and HF 30-day case fatality rate, by age				
<55	0.1455	n/a	n/a	Spencer et al.[45]
55-64	0.1707	n/a	n/a	
65-74	0.2202	n/a	n/a	
75-84	0.2663	n/a	n/a	
>85	0.2959	n/a	n/a	
Mortality Rates				

© 2025 Kohli-Lynch CN et al. *JAMA Cardiology*.

Model Input	Mean	Standard Deviation	Distribution	Source
CHD – Angina (30-day mortality), by age				
35-44	0.0103	n/a	n/a	Bryant et al.[2] Bryant et al.[7] Bryan et al.[6]
45-54	0.0150	n/a	n/a	
55-64	0.0196	n/a	n/a	
65-74	0.0242	n/a	n/a	
75-84	0.0332	n/a	n/a	
>85	0.0559	n/a	n/a	
CHD – MI, Primary (30-day mortality), by age				
Male				
35-44	0.0302	n/a	n/a	Bryant et al.[2] Bryant et al.[7] Bryan et al.[6]
45-54	0.0307	n/a	n/a	
55-64	0.0541	n/a	n/a	
65-74	0.0860	n/a	n/a	
75-84	0.1500	n/a	n/a	
>85	0.2000	n/a	n/a	
Female				
35-44	0.0302	n/a	n/a	Bryant et al.[2] Bryant et al.[7] Bryan et al.[6]
45-54	0.0455	n/a	n/a	
55-64	0.0800	n/a	n/a	
65-74	0.1093	n/a	n/a	
75-84	0.1186	n/a	n/a	
>85	0.1786	n/a	n/a	
CHD – MI, Recurrent (30-day mortality), by age				
Male				
35-44	0.0484	n/a	n/a	Bryant et al.[2] Bryant et al.[7] Bryan et al.[6]
45-54	0.0491	n/a	n/a	
55-64	0.0866	n/a	n/a	
65-74	0.1376	n/a	n/a	
75-84	0.1816	n/a	n/a	
>85	0.2858	n/a	n/a	
Female				
35-44	0.0483	n/a	n/a	Bryant et al.[2] Bryant et al.[7] Bryan et al.[6]
45-54	0.0728	n/a	n/a	
55-64	0.1279	n/a	n/a	
65-74	0.1748	n/a	n/a	
75-84	0.1819	n/a	n/a	
>85	0.2411	n/a	n/a	
CHD – cardiac arrest (survive to hospital), by age				
35-44	0.1537	n/a	n/a	Bryant et al.[2] Bryant et al.[7] Bryan et al.[6]
45-54	0.0684	n/a	n/a	
55-64	0.0508	n/a	n/a	
65-74	0.0753	n/a	n/a	
75-84	0.0672	n/a	n/a	

© 2025 Kohli-Lynch CN et al. *JAMA Cardiology*.

Model Input	Mean	Standard Deviation	Distribution	Source
>85	0.0424	n/a	n/a	
CHD – cardiac arrest (30-day survival post hospital), by age				
35-44	0.0113	n/a	n/a	Bryant et al.[2] Bryant et al.[7] Bryan et al.[6]
45-54	0.0389	n/a	n/a	
55-64	0.1154	n/a	n/a	
65-74	0.0838	n/a	n/a	
75-84	0.0342	n/a	n/a	
>85	0.0342	n/a	n/a	
HF (30-day mortality), by age				
35-44	0.0353	n/a	n/a	AHRQ HCUP[50] Krumholz et al.[51]
45-54	0.0353	n/a	n/a	
55-64	0.0421	n/a	n/a	
65-74	0.0598	n/a	n/a	
75-84	0.0776	n/a	n/a	
>85	0.1440	n/a	n/a	
Stroke (initial hospitalization mortality), by age				
Male				
35-44	0.0542	n/a	n/a	Bryant et al.[2] Bryant et al.[7] Bryan et al.[6]
45-54	0.0516	n/a	n/a	
55-64	0.0462	n/a	n/a	
65-74	0.0458	n/a	n/a	
75-84	0.0625	n/a	n/a	
>85	0.0922	n/a	n/a	
Female				
35-44	0.0444	n/a	n/a	Bryant et al.[2] Bryant et al.[7] Bryan et al.[6]
45-54	0.0508	n/a	n/a	
55-64	0.0457	n/a	n/a	
65-74	0.0471	n/a	n/a	
75-84	0.0696	n/a	n/a	
>85	0.0947	n/a	n/a	
Stroke (1-year mortality), by age				
Male				
35-44	0.0795	n/a	n/a	Bryant et al.[2] Bryant et al.[7] Bryan et al.[6]
45-54	0.0800	n/a	n/a	
55-64	0.0850	n/a	n/a	
65-74	0.0900	n/a	n/a	
75-84	0.1375	n/a	n/a	
>85	0.2650	n/a	n/a	
Female				
35-44	0.0574	n/a	n/a	Bryant et al.[2] Bryant et al.[7] Bryan et al.[6]
45-54	0.0630	n/a	n/a	
55-64	0.0630	n/a	n/a	
65-74	0.0700	n/a	n/a	

© 2025 Kohli-Lynch CN et al. *JAMA Cardiology*.

Model Input	Mean	Standard Deviation	Distribution	Source
75-84	0.1100	n/a	n/a	
>85	0.2700	n/a	n/a	
CABG operative mortality				
Male				
35-44	0.0082	n/a	n/a	Bryant et al.[2] Bryant et al.[7] Bryan et al.[6]
45-54	0.0054	n/a	n/a	
55-64	0.0054	n/a	n/a	
65-74	0.0144	n/a	n/a	
75-84	0.0270	n/a	n/a	
>85	0.1196	n/a	n/a	
Female				
35-44	0.0413	n/a	n/a	Bryant et al.[2] Bryant et al.[7] Bryan et al.[6]
45-54	0.0413	n/a	n/a	
55-64	0.0172	n/a	n/a	
65-74	0.0107	n/a	n/a	
75-84	0.0561	n/a	n/a	
>85	0.1675	n/a	n/a	
PTCA case fatality rate				
Male				
35-44	0.0000	n/a	n/a	Bryant et al.[2] Bryant et al.[7] Bryan et al.[6]
45-54	0.0006	n/a	n/a	
55-64	0.0004	n/a	n/a	
65-74	0.0029	n/a	n/a	
75-84	0.0056	n/a	n/a	
>85	0.0044	n/a	n/a	
Female				
35-44	0.0000	n/a	n/a	Bryant et al.[2] Bryant et al.[7] Bryan et al.[6]
45-54	0.0037	n/a	n/a	
55-64	0.0026	n/a	n/a	
65-74	0.0054	n/a	n/a	
75-84	0.0196	n/a	n/a	
>85	0.0268	n/a	n/a	

BMI – Body Mass Index, CA – Cardiac Arrest, CABG – Coronary Artery Bypass Graft, CU-NHLBI PCS – Columbia University-NHLBI Pooled Cohorts Study, CVD – Cardiovascular Disease, DM – Diabetes Mellitus, eGFR – Estimated Glomerular Filtration Rate, HDL-C – High-Density Lipoprotein Cholesterol, HF – Heart Failure, LDL-C – Low-Density Lipoprotein Cholesterol, MI – Myocardial Infarction, PTCA – Percutaneous Transluminal Coronary Angioplasty, SBP – Systolic Blood Pressure, SD – Standard Deviation

© 2025 Kohli-Lynch CN et al. *JAMA Cardiology.*

eTable 4. Health-related costs and utility values in CVDPM

Model Input	Mean	SD	Min	Max	Distribution	Source
Costs						
Office visits						
Routine visit	75.32	6.92	68.81	96.49	Gamma	CMS Physician Fee Schedule[52]
Intolerable AE	110.28	10.31	101.17	142.41	Gamma	
Serious AE	147.76	14.06	135.73	191.96	Gamma	
Chronic (non hospitalization) health state costs						
CHD <70<br>Year after event	13,273	-	-	-	-	Regression analysis in MEPS[31]
CHD ≥70<br>Year after event	20,284	-	-	-	-	
Stroke <70<br>Year after event	18,557	-	-	-	-	
Stroke ≥70<br>Year After events	18,557	-	-	-	-	
Background health state costs	Vary according to age, sex, race, selected comorbidities, history of CVD events, and long-term care					
Non-MI ACS hospitalization, non-fatal						
18-34	14,087	665	13,098	15,038	Gamma	NIS[37]<br>Arth et al.[38]<br>Peterson et al.[39]
35-44	18,121	622	17,625	18,602	Gamma	
45-54	20,075	653	19,777	20,402	Gamma	
55-64	22,796	733	22,513	23,078	Gamma	
65-74	24,492	786	24,218	24,771	Gamma	
74-84	23,706	768	23,355	24,040	Gamma	
84+	17,543	604	17,090	18,030	Gamma	
Non-MI ACS hospitalization, fatal						
18-64	67,116	9,236	49,853	85,141	Gamma	NIS[37]<br>Arth et al.[38]<br>Peterson et al.[39]
65-74	65,724	4,072	58,963	72,451	Gamma	
74-84	55,990	3,411	50,495	62,037	Gamma	
84+	32,420	4,107	24,942	40,602	Gamma	
Cardiac arrest hospitalization, non-fatal						
18-54	30,878	3,621	24,209	37,621	Gamma	NIS[37]<br>Arth et al.[38]<br>Peterson et al.[39]
55-64	31,695	2,661	26,941	36,802	Gamma	
65-74	33,876	2,923	28,518	39,057	Gamma	
74-84	25,360	1,772	22,379	28,707	Gamma	
84+	11,884	907	10,300	13,565	Gamma	
Cardiac arrest hospitalization, fatal						
18-34	16,886	1,255	14,840	19,248	Gamma	NIS[37]<br>Arth et al.[38]<br>Peterson et al.[39]
35-44	16,755	1,103	14,839	18,637	Gamma	
45-54	15,835	827	14,602	17,210	Gamma	
55-64	13,929	623	13,085	14,798	Gamma	
65-74	12,422	574	11,592	13,265	Gamma	
74-84	10,678	499	10,028	11,465	Gamma	
84+	8,709	495	7,930	9,509	Gamma	

© 2025 Kohli-Lynch CN et al. *JAMA Cardiology.*

Model Input	Mean	SD	Min	Max	Distribution	Source
HF hospitalization, non-fatal						
18-34	20,314	1,164	18,386	22,355	Gamma	NIS[37] Arth et al.[38] Peterson et al.[39]
35-44	15,563	637	14,740	16,372	Gamma	
45-54	14,753	544	14,229	15,328	Gamma	
55-64	15,134	522	14,732	15,563	Gamma	
65-74	13,973	465	13,679	14,272	Gamma	
74-84	11,861	383	11,703	12,032	Gamma	
84+	9,936	319	9,816	10,058	Gamma	
HF hospitalization, fatal						
18-44	101,719	16,926	73,487	138,401	Gamma	NIS[37] Arth et al.[38] Peterson et al.[39]
45-54	63,123	6,859	51,169	76,047	Gamma	
55-64	55,783	3,919	49,352	63,248	Gamma	
65-74	36,391	2,055	33,096	39,893	Gamma	
74-84	23,928	1,143	22,307	25,604	Gamma	
84+	15,456	669	14,481	16,350	Gamma	
MI hospitalization, non-fatal						
18-34	18,341	766	17,360	19,363	Gamma	NIS[37] Arth et al.[38] Peterson et al.[39]
35-44	20,885	691	20,480	21,303	Gamma	
45-54	22,107	710	21,844	22,363	Gamma	
55-64	23,724	761	23,450	23,997	Gamma	
65-74	23,951	769	23,684	24,231	Gamma	
74-84	22,058	711	21,768	22,357	Gamma	
84+	15,782	516	15,507	16,050	Gamma	
MI hospitalization, fatal						
18-44	47,092	6,373	35,523	59,965	Gamma	NIS[37] Arth et al.[38] Peterson et al.[39]
45-54	40,837	2,203	37,393	44,648	Gamma	
55-64	38,593	1,695	36,243	40,860	Gamma	
65-74	35,285	1,462	33,486	37,143	Gamma	
74-84	28,902	1,183	27,370	30,295	Gamma	
84+	16,131	775	14,975	17,304	Gamma	
Stroke hospitalization, non-fatal						
18-34	27,462	1,144	26,013	28,939	Gamma	NIS[37] Arth et al.[38] Peterson et al.[39]
35-44	23,109	870	22,204	24,055	Gamma	
45-54	18,961	643	18,494	19,432	Gamma	
55-64	16,611	549	16,273	16,932	Gamma	
65-74	14,286	466	14,062	14,528	Gamma	
74-84	13,151	424	12,971	13,320	Gamma	
84+	11,900	386	11,731	12,078	Gamma	
Stroke hospitalization, fatal						
18-34	36,824	5,401	26,714	47,086	Gamma	NIS[37] Arth et al.[38] Peterson et al.[39]
35-44	32,382	2,202	28,550	36,258	Gamma	
45-54	29,399	1,376	27,310	31,556	Gamma	
55-64	26,944	1,130	25,488	28,420	Gamma	
65-74	22,964	934	21,910	24,138	Gamma	

© 2025 Kohli-Lynch CN et al. *JAMA Cardiology*.

Model Input	Mean	SD	Min	Max	Distribution	Source
74-84	18,903	763	18,015	19,933	Gamma	
84+	13,576	557	12,904	14,283	Gamma	
Long-term care costs						
Men <65	87	1	85	90	Gamma	Howden et al.[33] Harris-Kotjetin et al.[32]
Men 65-74	839	11	818	861	Gamma	
Men 75-84	2,851	38	2,776	2,927	Gamma	
Men ≥85	13,480	183	13,115	13,845	Gamma	
Women <65	171	2	167	176	Gamma	
Women 65-74	1,482	20	1,443	1,521	Gamma	
Women 75-84	4,255	57	4,141	4,370	Gamma	
Women ≥85	13,620	187	13,247	13,993	Gamma	
ESRD annual cost	91,645	3,933	79,392	94,811	Gamma	U.S. Renal Data System[53]
Utilities						
Adverse events						
Intolerable[a]	0.2000	0.03	0.17	0.23	Beta	Bryant et al.[2]
Serious[b]	0.1000	0.03	0.08	0.13	Beta	Bress et al.[54]
Acute CVD event[b]						
Angina	0.0936	0.0150	0.0636	0.1236	Beta	Moran et al.[40] Moran et al.[41] Murray et al.[42]
Cardiac arrest	0.0948	0.0150	0.0648	0.2340	Beta	Moran et al.[40] Moran et al.[41] Murray et al.[42]
HF	0.1000	0.0150	0.0700	0.1300	Beta	King et al.[55] Bress et al.[54]
MI	0.0948	0.0150	0.0648	0.2340	Beta	Moran et al.[40] Moran et al.[41] Murray et al.[42]
Stroke	0.1356	0.1056	0.1056	0.1356	Beta	Moran et al.[40] Moran et al.[41] Murray et al.[42]
Chronic utilities						
Intercept	0.9442	-	-	-	-	Sullivan et al.[56]
Age	-0.0007	-	-	-	-	
Male sex	0.0007	-	-	-	-	
Obese	-0.0500	-	-	-	-	
# of comorbidities	-0.0546	-	-	-	-	
# of comorbidities squared	0.0031	-	-	-	-	
Angina	-0.0412	-	-	-	-	
Cardiac arrest	-0.0190	-	-	-	-	
HF	-0.0635	-	-	-	-	
MI	-0.0409	-	-	-	-	
Stroke	-0.0524	-	-	-	-	

© 2025 Kohli-Lynch CN et al. *JAMA Cardiology*.

Model Input	Mean	SD	Min	Max	Distribution	Source
Diabetes	-0.0351	-	-	-	-	
ESRD	-0.0603	-	-	-	-	
Hyperlipidemia	-0.0049	-	-	-	-	
Hypertension	-0.0250	-	-	-	-	
Pill-taking disutility	0.0020	0.0008	0.0000	0.0083	Beta	Kohli-Lynch et al.[1] Hutchins et al.[57] Hutchins et al.[58]

ACS – Acute Coronary Syndrome, BP – Blood Pressure, CHD – Coronary Heart Disease, CMS – Centers for Medicare and Medicaid Services, CVD – Cardiovascular Disease, DBP – Diastolic Blood Pressure, ESRD – End-Stage Renal Disease, FPL – Federal Poverty Level, HF – Heart Failure, LDL-C – Low-Density Lipoprotein Cholesterol, MEPS – Medical Expenditure Panel Survey, MI – Myocardial Infarction, SBP – Systolic Blood Pressure, SD – Standard Deviation

[a]Applied for two days
[b]Applied for up to four weeks

© 2025 Kohli-Lynch CN et al. *JAMA Cardiology*.

eTable 5. Validation of one-year blood pressure and lipid outcomes vs polypill trial

Outcome	Polypill Trial	Simulated Outcomes (95% UI)
Systolic Blood Pressure (mm Hg)		
Baseline	140	140.2 (140.1-140.3)
Usual Care, after 1 year	138	139.6 (139.5-139.7)
Polypill, after 1 year	131	131.9 (131.5-132.0)
Difference, after 1 year	7.0	7.6 (7.5-7.9)
LDL Cholesterol (mg/dL)		
Baseline	113	113.5 (113.3-113.7)
Usual Care, after 1 year	109	109.7 (109.5-109.8)
Polypill, after 1 year	98	98.4 (98.3-98.6)
Difference, after 1 year	11	11.2 (11.1-11.3)

LDL – Low-Density Lipoprotein, UI – Uncertainty Interval.

Notes: The table shows one-year outcomes of the SCCS polypill trial compared with the simulated outcomes. The simulated outcomes values are the mean and 95% UI (2.5th to 97.5th percentiles of means) from 100 probabilistic runs the model.

© 2025 Kohli-Lynch CN et al. *JAMA Cardiology*.

eTable 6. Modeled SBP and LDL-C reductions, risk adjustments, and adverse events

Model Input	Mean	Standard Deviation	Min	Max	Distribution	Source
Blood pressure medications parameters						
Blood pressure change with medication: per full standard dose added						
Mean DBP reduction at 90 mmHg	4.700	0.421	2.350	7.050	Gamma	Law, Morris, and Wald.[27] Law et al.[13]
Coefficient of reduction per mmHg decrease in pretreatment DBP	0.110	0.028	0.055	0.165	Gamma	
Mean SBP reduction at 150 mmHg	8.700	0.357	4.350	13.050	Gamma	
Coefficient of reduction per mmHg decrease in pretreatment SBP	0.100	0.025	0.050	0.150	Gamma	
Blood pressure change with medication: per half standard dose added						
Mean DBP reduction at 90 mmHg	3.700	0.306	3.100	4.300	Gamma	Law, Morris, and Wald.[27] Law et al.[13]
Coefficient of reduction per mmHg decrease in pretreatment DBP	0.088	0.022	0.045	0.132	Gamma	
Mean SBP reduction at 150 mmHg	6.700	0.281	6.100	7.200	Gamma	
Coefficient of reduction per mmHg decrease in pretreatment SBP	0.078	0.020	0.039	0.117	Gamma	
Blood pressure change with medication: per two half standard doses						
Mean DBP reduction at 90 mmHg	7.300	0.536	6.200	8.300	Gamma	Law et al.[13]
Coefficient of reduction per mmHg decrease in pretreatment DBP (multiplied by 2 medications)	0.088	0.022	0.045	0.132	Gamma	
Mean SBP reduction at 150 mmHg	13.300	0.434	12.400	14.100	Gamma	
Coefficient of reduction per mmHg decrease in pretreatment SBP (multiplied by 2 medications)	0.078	0.020	0.039	0.117	Gamma	
Risk adjustment attributable to reduced blood pressure						
Relative risk of CVD events per 10 mmHg reduction in SBP						
CHD	0.830	-	0.780	0.880	Lognormal	Ettehad et al.[10]
HF	0.720	-	0.670	0.780	Lognormal	

© 2025 Kohli-Lynch CN et al. *JAMA Cardiology*.

Model Input	Mean	Standard Deviation	Min	Max	Distribution	Source
Stroke	0.730	-	0.680	0.770	Lognormal	
Hazard ratio of mortality risk with HF						
CVD mortality	2.94	-	2.41	3.58	Lognormal	Gerber et al.[48]
Non-CVD Mortality	2.10	-	1.74	2.55	Lognormal	
Adverse events with blood pressure medication						
Any adverse events, by number of standard dose medications						
1 half	0.031	0.024	0.001	0.093	Beta	Law, Morris, and Wald.[27] Law et al.[13] NHANES[59]
1 full	0.055	0.018	0.024	0.096	Beta	
1 full + 1 half	0.066	0.010	0.055	0.096	Beta	
2 full	0.087	0.008	0.073	0.103	Beta	
2 full + 1 half	0.101	0.010	0.087	0.125	Beta	
3 full	0.117	0.008	0.103	0.134	Beta	
3 full + 1 half	0.130	0.009	0.117	0.154	Beta	
4 full	0.147	0.008	0.133	0.164	Beta	
4 full + 1 half	0.161	0.010	0.147	0.186	Beta	
5 full	0.177	0.008	0.162	0.194	Beta	
Intolerable adverse events, by number of antihypertensive medication classes						
1 class	0.005	0.002	0.001	0.011	Beta	Law, Morris, and Wald.[27] Law et al.[13] NHANES[59]
2 classes	0.009	0.005	0.003	0.021	Beta	
3 classes	0.016	0.008	0.005	0.035	Beta	
4 classes	0.023	0.011	0.007	0.050	Beta	
5 classes	0.030	0.014	0.009	0.064	Beta	
Serious adverse events, by number of antihypertensive medication classes						
≤2 classes	0.009	0.001	0.006	0.010	Beta	Xie et al.[15]

© 2025 Kohli-Lynch CN et al. *JAMA Cardiology*.

Model Input	Mean	Standard Deviation	Min	Max	Distribution	Source
>2 classes	0.013	0.002	0.011	0.017	Beta	Wright et al.[14]
Probability serious adverse event is fatal, by age						
18-44	0.004	0.001	0.004	0.007	Beta	AHRQ HCUP[50] Wright et al.[14] Bress et al.[54]
45-64	0.011	0.003	0.005	0.017	Beta	
65-84	0.019	0.007	0.003	0.031	Beta	
≥85	0.031	0.010	0.015	0.053	Beta	
Blood pressure medication adherence, by number of medication classes						
1 class	0.900	0.070	0.681	0.963	Beta	Claxton et al.[25] Vrijens et al.[22]
2 classes	0.845	0.057	0.700	0.927	Beta	
3 classes	0.823	0.055	0.712	0.932	Beta	
≥4 classes	0.747	0.053	0.669	0.881	Beta	
BP medication discontinuation within 1 year of initiation	0.430	0.049	0.340	0.535	Weibull	Bellows et al.[3]
LDL-C reducing medication parameters						
LDL-C reduction with full adherence (%)**	37.1	4.7	27.8	46.3	Normal	Adams et al.[29]
Relative risk of CVD events per 1.0 mmol/L (38.67 mg/dL) LDL-C reduction						
CHD	0.76	0.015	0.73	0.79	Normal	Mihaylova et al.[11]
Stroke	0.85	0.023	0.80	0.89	Normal	
Adverse events, annual probability						
Any adverse event	0.0050	0.0013	0.0024	0.0076	Beta	Cai et al.[16] Newman et al.[60]
Intolerable adverse event	0.0010	0.0151	0.0893	0.1488	Beta	
Serious adverse event	0.0001	0.00002	0.00008	0.00014	Beta	
Statin-induced diabetes	0.0050	0.0023	0.0010	0.010	Beta	Finegold et al.[61]
Adherence	0.792	0.078	0.694	1.000	Beta	Recalibrated

© 2025 Kohli-Lynch CN et al. *JAMA Cardiology*.

Model Input	Mean	Standard Deviation	Min	Max	Distribution	Source
Probability of discontinuation within one year of initiation	0.430	0.049	0.340	0.535	Weibull	Bellows et al.[3]
Polypill medication parameters						
Adherence	0.860	0.025	0.810	0.910	Beta	Muñoz et al.[24]
Probability of discontinuation within one year of initiation	0.220	0.028	0.165	0.275	Weibull	Muñoz et al.[24]

BP – Blood Pressure, CHD – Coronary Heart Disease, CVD – Cardiovascular Disease, DBP – Diastolic Blood Pressure, HF – Heart Failure, LDL-C – Low-Density Lipoprotein Cholesterol, SBP – Systolic Blood Pressure, SD – Standard Deviation

© 2025 Kohli-Lynch CN et al. *JAMA Cardiology*.

eTable 7. Medication prices (USD 2023)

Model Input	Value	SD	Min	Max	Distribution	Source
Base Case: MEPS Median, Utilization-Weighted						
BP medication costs, annual ($)						
1 half	117	8	101	133	Gamma	MEPS[31]
1 full	122	9	104	139	Gamma	
1 full + 1 half	205	13	180	230	Gamma	
2 full	210	13	184	236	Gamma	
2 full + 1 half	357	20	317	397	Gamma	
3 full	358	21	317	400	Gamma	
3 full + 1 half	509	51	410	609	Gamma	
4 full	515	54	408	621	Gamma	
4 full + 1 half	563	52	462	665	Gamma	
5 full	581	52	480	683	Gamma	
Other medication costs, annual ($)						
Intermediate-intensity statin therapy	112	31	41	164	Gamma	MEPS[31]
Polypill medication[a]	463	n/a	349	577	Gamma	Assumption[b]
Pricing Scenario 1: MEPS Mean, Utilization-Weighted						
BP medication costs, annual ($)						
1 half	212	n/a			Gamma	MEPS[31]
1 full	244	n/a			Gamma	
1 full + 1 half	433	n/a			Gamma	
2 full	462	n/a			Gamma	
2 full + 1 half	677	n/a			Gamma	
3 full	709	n/a			Gamma	
3 full + 1 half	984	n/a			Gamma	
4 full	1,025	n/a			Gamma	
4 full + 1 half	1,047	n/a			Gamma	
5 full	1,050	n/a			Gamma	
Other medication costs, annual ($)						
Intermediate-intensity statin therapy	238	n/a			Gamma	MEPS[31]
Polypill medication[a]	872	n/a			Gamma	Assumption[b]
Pricing Scenario 2: National Average Drug Acquisition Cost[c]						

© 2025 Kohli-Lynch CN et al. *JAMA Cardiology*.

BP medication costs, annual ($)				
Amlodipine 2.5 mg	48	n/a	Gamma	NADAC[35]
Losartan 25 mg	59	n/a	Gamma	
HCTZ 12.5 mg	61	n/a	Gamma	
Other medication costs, annual ($)				
Atorvastatin 10 mg	54	n/a	Gamma	NADAC[35]
Polypill	222	n/a	Gamma	Assumption[b]

BP – Blood Pressure, HCTZ – Hydrochlorothiazide, MEPS – Medical Expenditure Panel Survey, NADAC – National Average Drug Acquisition Cost, SD – Standard Deviation

[a]Varied extensively in sensitivity analyses
[b]Combined price of component medications
[c]Nominal dispensing fee applied to all costs ($10.50 per prescription, assuming 90-day refills[36])

© 2025 Kohli-Lynch CN et al. *JAMA Cardiology*.

eTable 8. Consolidated Health Economics Evaluation Reporting Standards (CHEERS) 2022 checklist[62]

Item Description		Guidance for Reporting	Reported in section
**Title**			
Title	1	Identify the study as an economic evaluation and specify theinterventions being compared.	Title Page
**Abstract**			
Abstract	2	Provide a structured summary that highlights context, key methods,results and alternative analyses.	Pages 2-3
**Introduction**			
Background and objectives	3	Give the context for the study, the study question and its practicalrelevance for decision making in policy or practice.	Page 4
**Methods**			
Health economic analysis plan	4	Indicate whether a health economic analysis plan was developed and where available.	Page 4
Study population	5	Describe characteristics of the study population (such as age range, demographics, socioeconomic, or clinical characteristics).	Page 5<br>Figure S1<br>Table S1
Setting and location	6	Provide relevant contextual information that may influence findings.	Pages 4-5
Comparators	7	Describe the interventions or strategies being compared and why chosen.	Pages 4-5
Perspective	8	State the perspective(s) adopted by the study and why chosen.	Page 6
Time horizon	9	State the time horizon for the study and why appropriate.	Page 6
Discount rate	10	Report the discount rate(s) and reason chosen.	Page 6
Selection of outcomes	11	Describe what outcomes were used as the measure(s) of benefit(s)and harm(s).	Page 6
Measurement of outcomes	12	Describe how outcomes used to capture benefit(s) and harm(s)were measured.	Pages 5-7<br>Pages S4-S5<br>Figure 1
Valuation of outcomes	13	Describe the population and methods used to measure and value outcomes.	Page 6<br>Pages S7-S8<br>Table S4
Measurement and valuation of resources and costs	14	Describe how costs were valued.	Page 6<br>Page S7<br>Table S4
Currency, price date,and conversion	15	Report the dates of the estimated resource quantities and unit costs, plus thecurrency and year of conversion.	Page 6<br>Page S6<br>Table 1<br>Table S4, Table S7
Rationale and description of model	16	If modelling is used, describe in detail and why used. Report if the model is publicly available and where it can be accessed.	Page 5<br>Pages S3-S6<br>Tables S2-S7<br>Figure 1<br>Figure S2
Analytics and assumptions	17	Describe any methods for analysing or statistically transforming data, anyextrapolation methods, and approaches for validating any model used.	Pages 7-8<br>Pages S3-S6<br>Table S5<br>Figure S3

© 2025 Kohli-Lynch CN et al. *JAMA Cardiology*.

Characterizing heterogeneity	18	Describe any methods used for estimating how the results of the study vary for sub-groups.	Page 5<br>Page 8<br>Tables S10-S11
Characterizing distributional effects	19	Describe how impacts are distributed across different individuals or adjustments made to reflect priority populations.	Page 5<br>Table S12
Characterizing uncertainty	20	Describe methods to characterize any sources of uncertainty in the analysis.	Page 8<br>Tables S2-S4
Approach to engagement with patients and others affected by the study	21	Describe any approaches to engage patients or service recipients, the general public, communities, or stakeholders (e.g., clinicians or payers) in the design of the study.	Not included
**Results**			
Study parameters	22	Report all analytic inputs (e.g., values, ranges, references) including uncertainty or distributional assumptions.	Page 6<br>Pages 8-9<br>Table 1
Summary of main results	23	Report the mean values for the main categories of costs and outcomes of interest and summarise them in the most appropriate overall measure.	Pages 9-10<br>Figures 2-3<br>Table 2<br>Table S9
Effect of uncertainty	24	Describe how uncertainty about analytic judgments, inputs, or projections affect findings. Report the effect of choice of discount rate and time horizon, if applicable.	Pages 9-10<br>Figure 2<br>Figures S5-S9
Effect of engagement with patients and others affected by the study	25	Report on any difference patient/service recipient, general public, community, or stakeholder involvement made to the approach or findings of the study	Not included
**Discussion**			
Study findings, limitations, generalizability, and current knowledge	26	Report key findings, limitations, ethical or equity considerations not captured, and how these could impact patients, policy, or practice.	Pages 10-11
**Other relevant information**			
Source of funding	27	Describe how the study was funded and any role of the funder in the identification, design, conduct, and reporting of the analysis	Page 1
Conflicts of interest	28	Report authors conflicts of interest according to journal or International Committee of Medical Journal Editors requirements.	Reported in journal portal

© 2025 Kohli-Lynch CN et al. *JAMA Cardiology*.

eTable 9. Disaggregated economic and clinical outcomes, polypill population

Outcome	Usual Care			Polypill			Incremental (Polypill vs Usual Care)		
	Mean	95% UI		Mean	95% UI		Mean	95% UI	
		Lower	Upper		Lower	Upper		Lower	Upper
**Total Costs[a]**	9,022,294,890	8,995,824,430	9,045,412,670	9,032,447,242	9,011,510,334	9,053,567,045	10,152,352	-13,329,689	36,618,357
Background	7,721,985,998	7,707,802,172	7,737,993,005	7,731,228,253	7,717,416,816	7,743,101,645	9,242,255	-3,891,652	25,043,053
Physician visits	76,108,459	76,007,776	76,190,672	74,997,648	74,893,083	75,095,366	-1,110,809	-1,256,947	-983,189
Medication	103,561,325	102,922,941	104,192,118	240,310,489	239,287,253	241,256,560	136,749,164	136,067,360	137,498,504
Acute CVD	488,981,263	480,797,844	499,268,803	427,722,942	418,678,268	434,764,180	-61,258,321	-76,803,030	-49,681,963
Chronic CVD	629,305,844	619,553,045	639,912,017	544,389,875	535,829,598	551,950,718	-84,915,969	-99,252,318	-73,453,522
AEs	2,352,002	2,028,399	2,605,179	13,798,035	13,112,784	14,632,368	11,446,033	10,763,000	12,348,863
**Total QALYs[a]**	601,599	600,642	602,526	602,786	602,081	603,530	1,187	287	2,159
Background	608,789	607,856	609,713	609,304	608,612	610,017	515	-379	1,480
Pill-taking disutility	-887	-891	-883	-1,079	-1,083	-1,075	-191	-195	-189
Acute CVD	-154	-156	-152	-134	-135	-132	20	17	23
Chronic CVD	-6,148	-6,259	-6,048	-5,300	-5,387	-5,209	848	719	998
AEs	-1	-1	-1	-5	-6	-5	-5	-5	-4
**Total CVD Events**	21,964	21,679	22,347	19,225	18,959	19,477	-2,738	-3,246	-2,374
CHD events	8,923	8,729	9,098	7,925	7,764	8,104	-997	-1,216	-755
HF	5,729	5,539	5,942	5,063	4,895	5,227	-666	-930	-445
Stroke	7,311	7,137	7,457	6,237	6,088	6,385	-1,074	-1,287	-844
Incident CVD events	18,635	18,435	18,899	16,455	16,262	16,649	-2,179	-2,511	-1,903
**Other Events**									
Life years	930,415	929,103	931,725	931,262	930,205	932,403	847	-576	2,341
Intolerable AEs	114	93	134	1,050	990	1,117	936	873	1,003
Serious AEs	198	171	219	1,079	1,024	1,146	881	826	950
CABG	589	539	640	520	478	572	-68	-142	11
PTCA	1,843	1,761	1,943	1,636	1,560	1,715	-207	-343	-81
Physician Office Visits	1,039,177	1,037,712	1,040,372	1,024,296	1,022,811	1,025,711	-14,881	-16,993	-13,063

AE – Adverse Event, CABG – Coronary Artery Bypass Graft, CVD – Cardiovascular Disease, HF – Heart Failure, PTCA - Percutaneous Transluminal Coronary Angioplasty, QALY – Quality-Adjusted Life Year, UI – Uncertainty Interval

[a]Discounted 3.0% annually

© 2025 Kohli-Lynch CN et al. *JAMA Cardiology*.

eTable 10. Cost-effectiveness in scenario analyses. 100,000 simulated individuals.

Mean Outcomes	Polypill Trial Population		
	Usual Care	Polypill	Incremental, Polypill vs Usual Care
Treat subset of polypill trial with baseline medication use (n=1,511 in NHANES cohort)			
Costs ($000's)	9,536,220	9,546,815	10,595
QALYs	589,237	590,602	1,364
ICER ($/QALY)	n/a	n/a	7,767
CVD Events	19,324	15,793	-3,531
Life Years	928,223	929,019	796
Increased pill-taking disutility for ≥2 medications			
Costs ($000's)	9,022,295	9,032,447	10,152
QALYs	601,222	602,780	1,557
ICER ($/QALY)	n/a	n/a	6,519
CVD Events	21,964	19,225	-2,738
Life Years	930,415	931,262	847
Lifetime horizon			
Costs ($000's)	24,315,568	24,433,766	118,197
QALYs	1,155,021	1,168,043	13,022
ICER ($/QALY)	n/a	n/a	9,077
CVD Events	55,356	50,586	-4,770
Life Years	2,394,772	2,421,603	26,831

CVD – Cardiovascular Disease, ICER – Incremental Cost-Effectiveness Ratio, QALY – Quality-Adjusted Life Year, UI – Uncertainty Interval

© 2025 Kohli-Lynch CN et al. *JAMA Cardiology*.

eTable 11. Cost-effectiveness in non-Hispanic Black U.S. population that meets polypill trial eligibility criteria (n=3,602,427).

**Outcomes**	**Polypill Trial Population (95% UI)**		
	**Usual Care**	**Polypill**	**Incremental, Polypill vs Usual Care**
Discounted Costs ($000's)	321,103,034 (320,213,292-322,089,673)	321,627,775 (320,891,292-322,562,068)	524,741 (-438,285-1,720,149)
Discounted QALYs	21,966,267 (21,939,573-21,992,529)	22,005,317 (21,977,362-22,031,975)	39,050 (6,592-75,795)
ICER ($/QALY)	n/a	n/a	13,438 (Cost Saving-52,021)
Total CVD Events	773,081 (762,490-786,230)	682,372 (671,817-693,179)	-90,709 (-104,290- -77,776)
Life Years	33,930,323 (33,896,569-33,963,322)	33,963,538 (33,915,733-34,009,505)	33,214 (-19,741-92,510)
Probability polypill preferred strategy at:			
$50,000/QALY	97%		
$100,000/QALY	98%		
$150,000/QALY	98%		

CVD – Cardiovascular Disease, ICER – Incremental Cost-Effectiveness Ratio, QALY – Quality-Adjusted Life Year, UI – Uncertainty Interval

© 2025 Kohli-Lynch CN et al. *JAMA Cardiology*.

eTable 12. Extended cost-effectiveness results

Household Income to Poverty Ratio	Baseline 10-Year ASCVD Risk (%)	Number of Individuals		QALYs Gained		ICER ($/QALY)
		Sum	% of Total	Sum	% of Gains	
<1.5 (poorest)	11.1	38,788	38.8	419	33.8	14,598
1.5-3.0	11.8	29,502	29.5	558	45.1	Cost Saving
>3 (richest)	9.9	31,710	31.7	260	21.0	41,508

CET – Cost-Effectiveness Threshold, ICER – Incremental Cost-Effectiveness Ratio, NMB – Net Monetary Benefit, QALY – Quality-Adjusted Life Year

One deterministic run of model with 100,000 simulated non-Hispanic Black U.S. individuals that meet polypill trial eligibility criteria

© 2025 Kohli-Lynch CN et al. *JAMA Cardiology*.

Supplemental Figures

eFigure 1. Inclusion and exclusion criteria for NHANES population

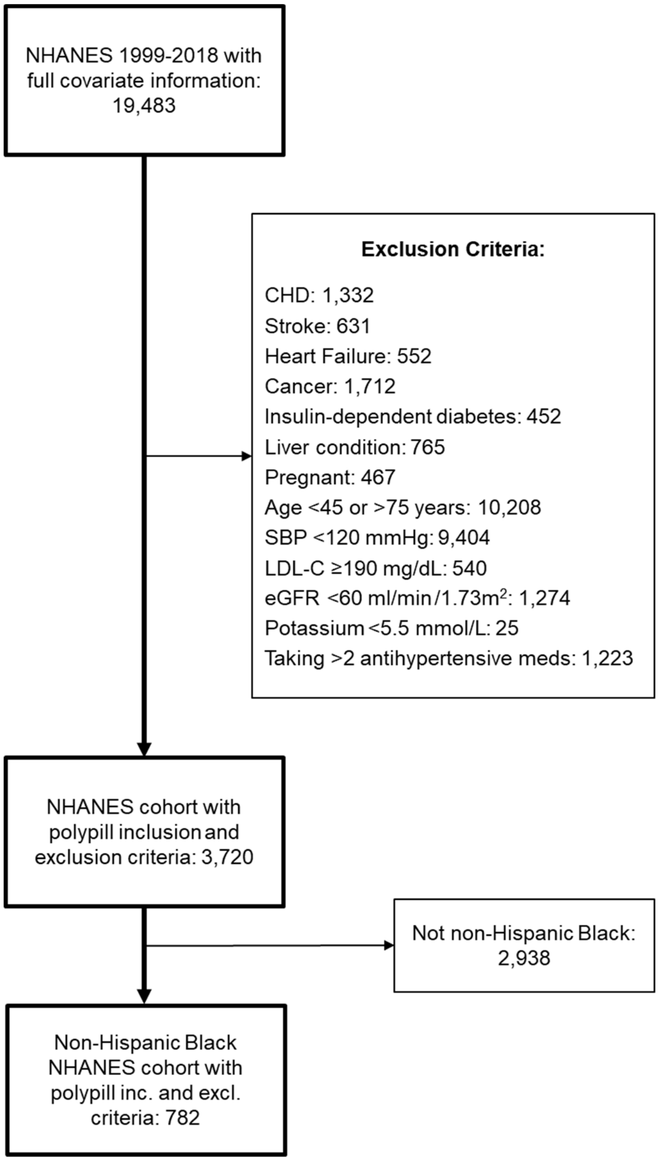

CHD – Coronary Heart Disease, eGFR – Estimated Glomerular Filtration Rate, LDL-C – Low-Density Lipoprotein Cholesterol, NHANES – National Health and Nutrition Examination Survey, SBP – Systolic Blood Pressure

© 2025 Kohli-Lynch CN et al. *JAMA Cardiology*.

eFigure 2. Discontinuation curve, polypill versus usual care (both BP and LDL-C medications)

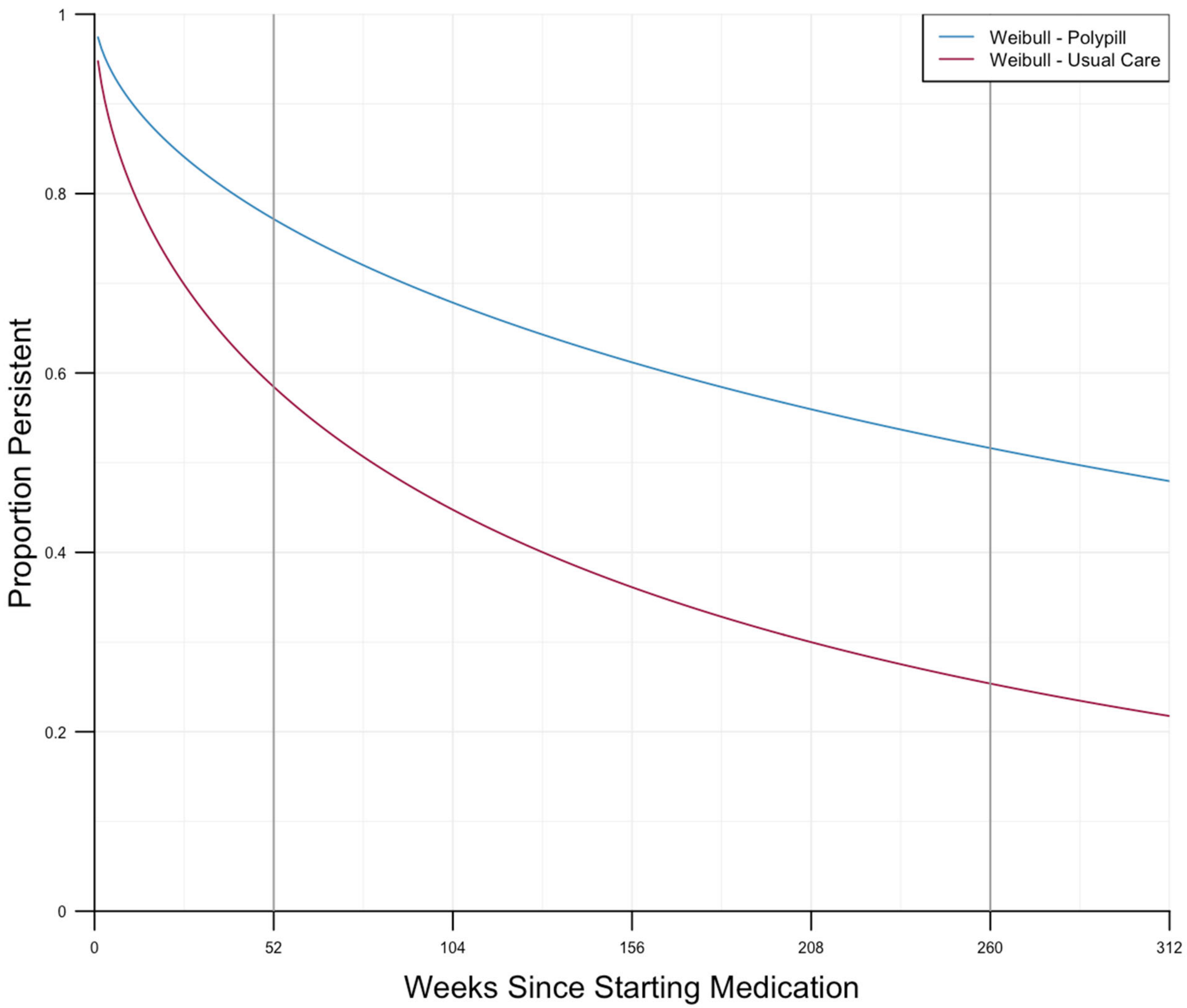

© 2025 Kohli-Lynch CN et al. *JAMA Cardiology*.

eFigure 3. Recalibration/Validation Figures, Women and Men: Death Rates, Total Event Rates, and Incident Event Rates

A. Recalibration/validation figures, women: death rates

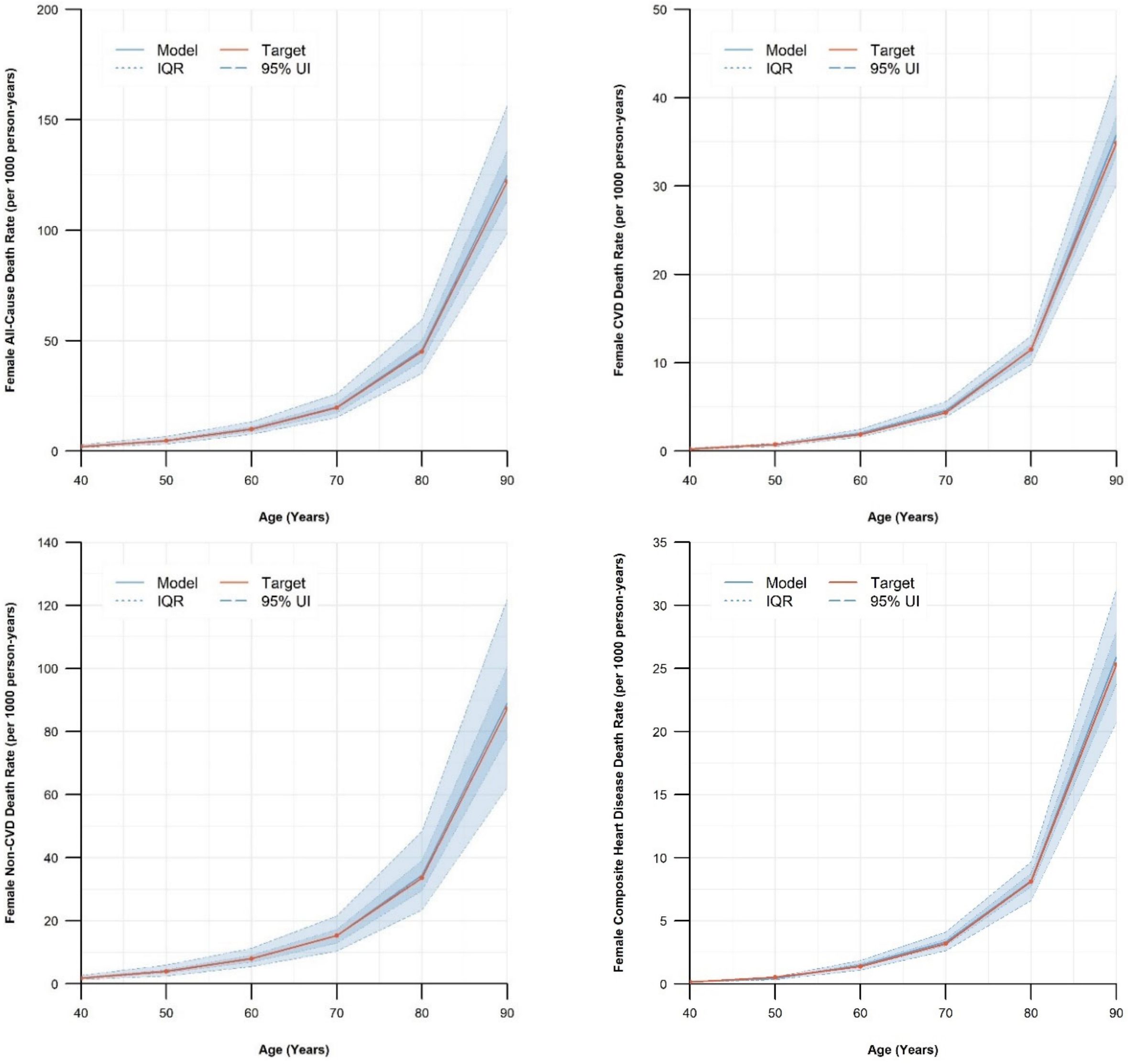

© 2025 Kohli-Lynch CN et al. *JAMA Cardiology*.

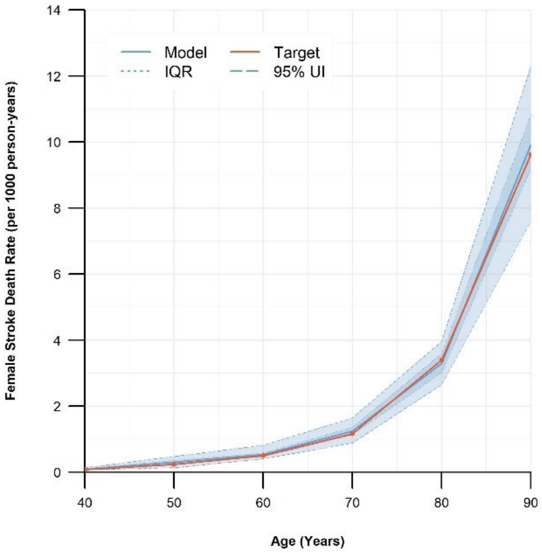

B. Recalibration/validation figures, women: total event rates

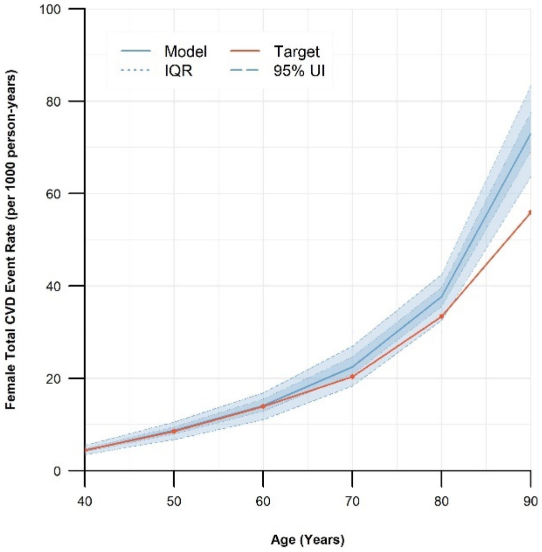

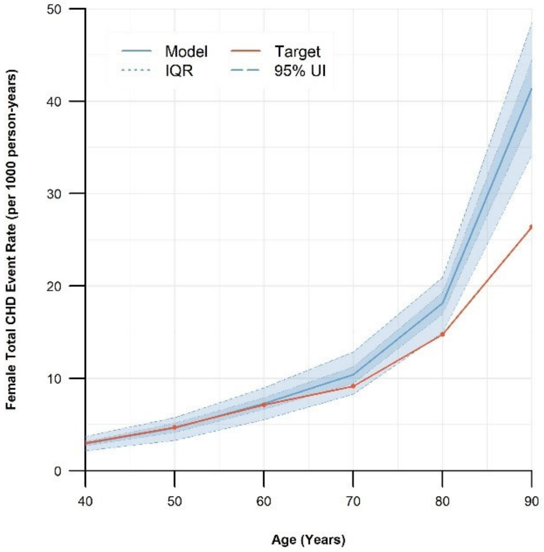

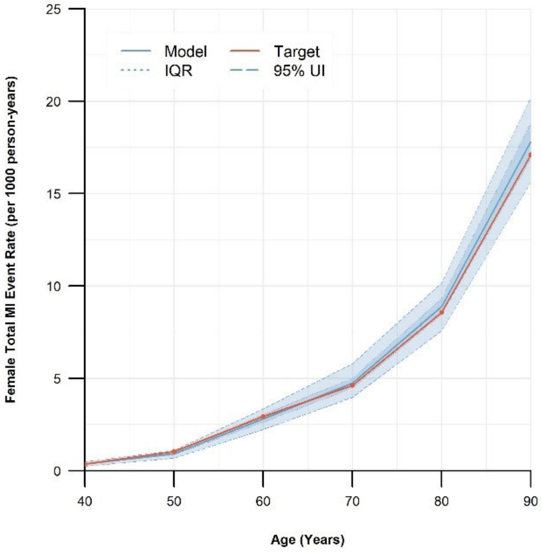

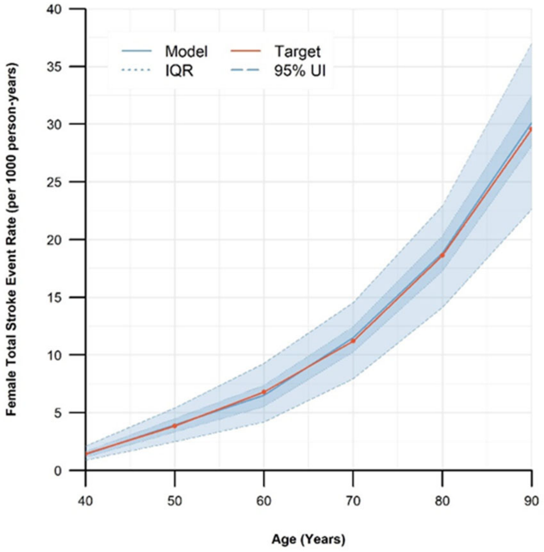

© 2025 Kohli-Lynch CN et al. *JAMA Cardiology*.

C. Recalibration/validation figures, women: incident event rates

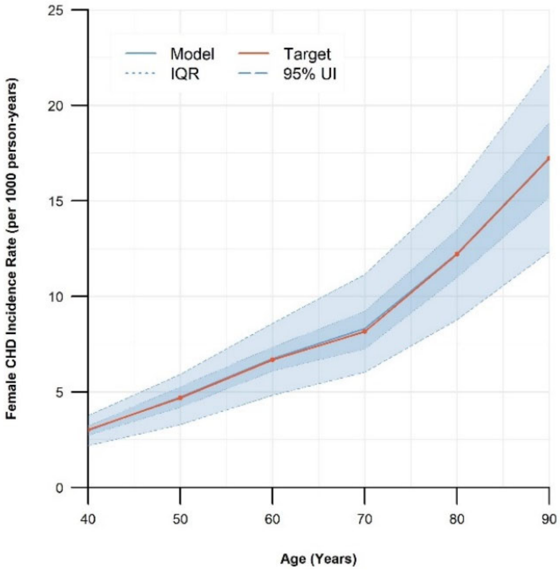

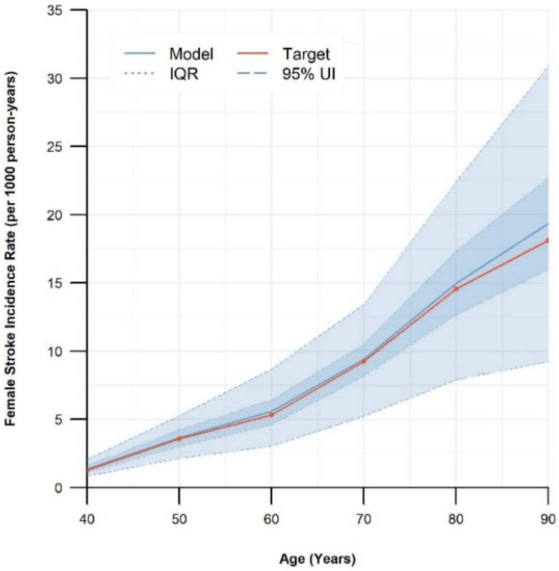

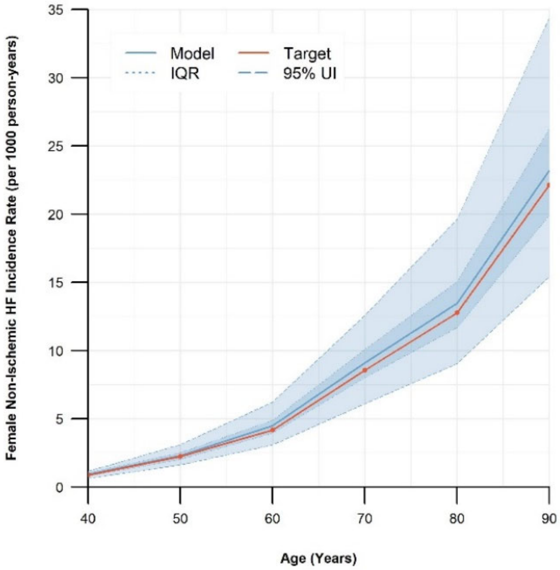

© 2025 Kohli-Lynch CN et al. *JAMA Cardiology*.

D. Recalibration/validation figures, men: death rates

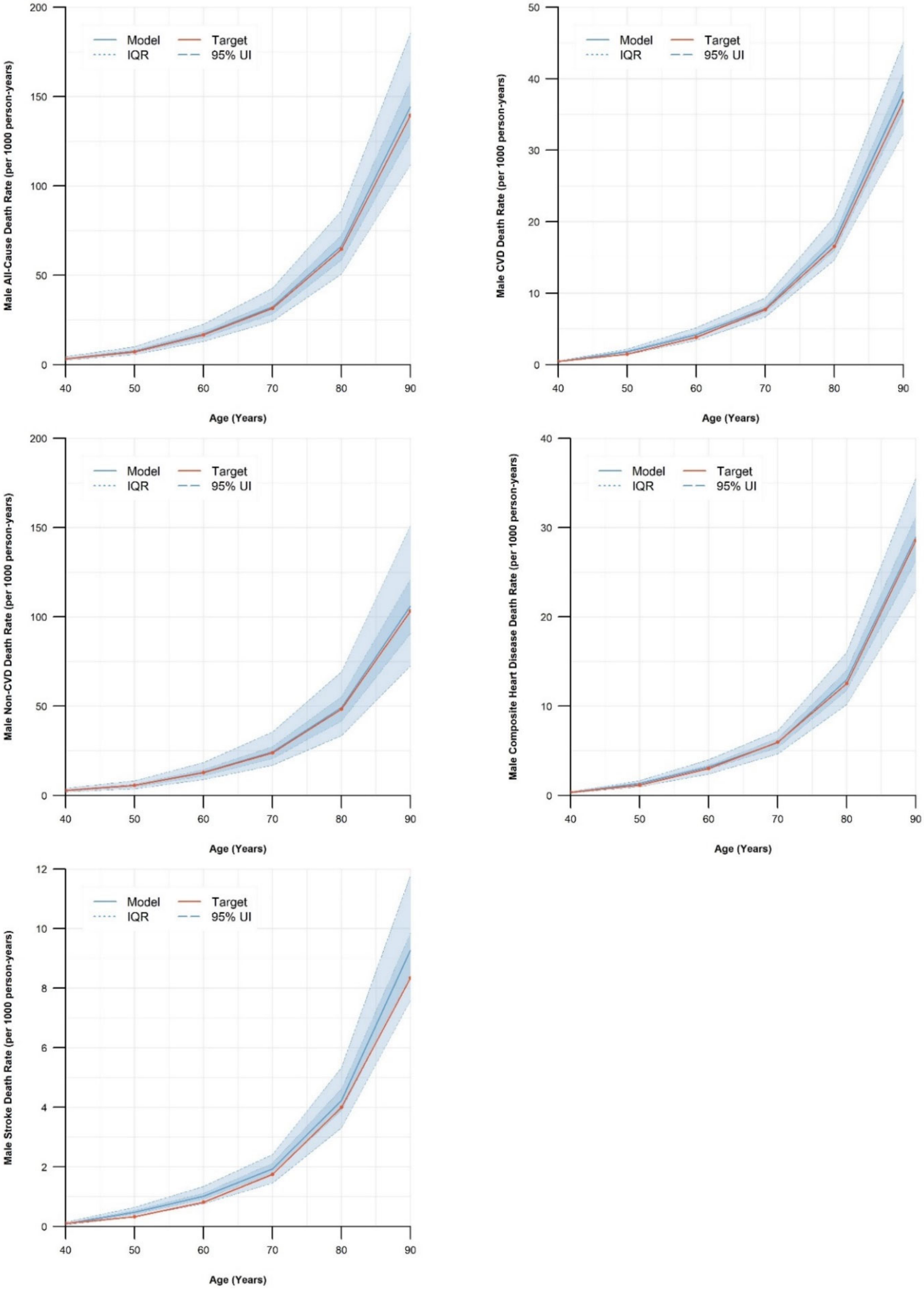

© 2025 Kohli-Lynch CN et al. *JAMA Cardiology*.

E. Recalibration/validation figures, men: total event rates

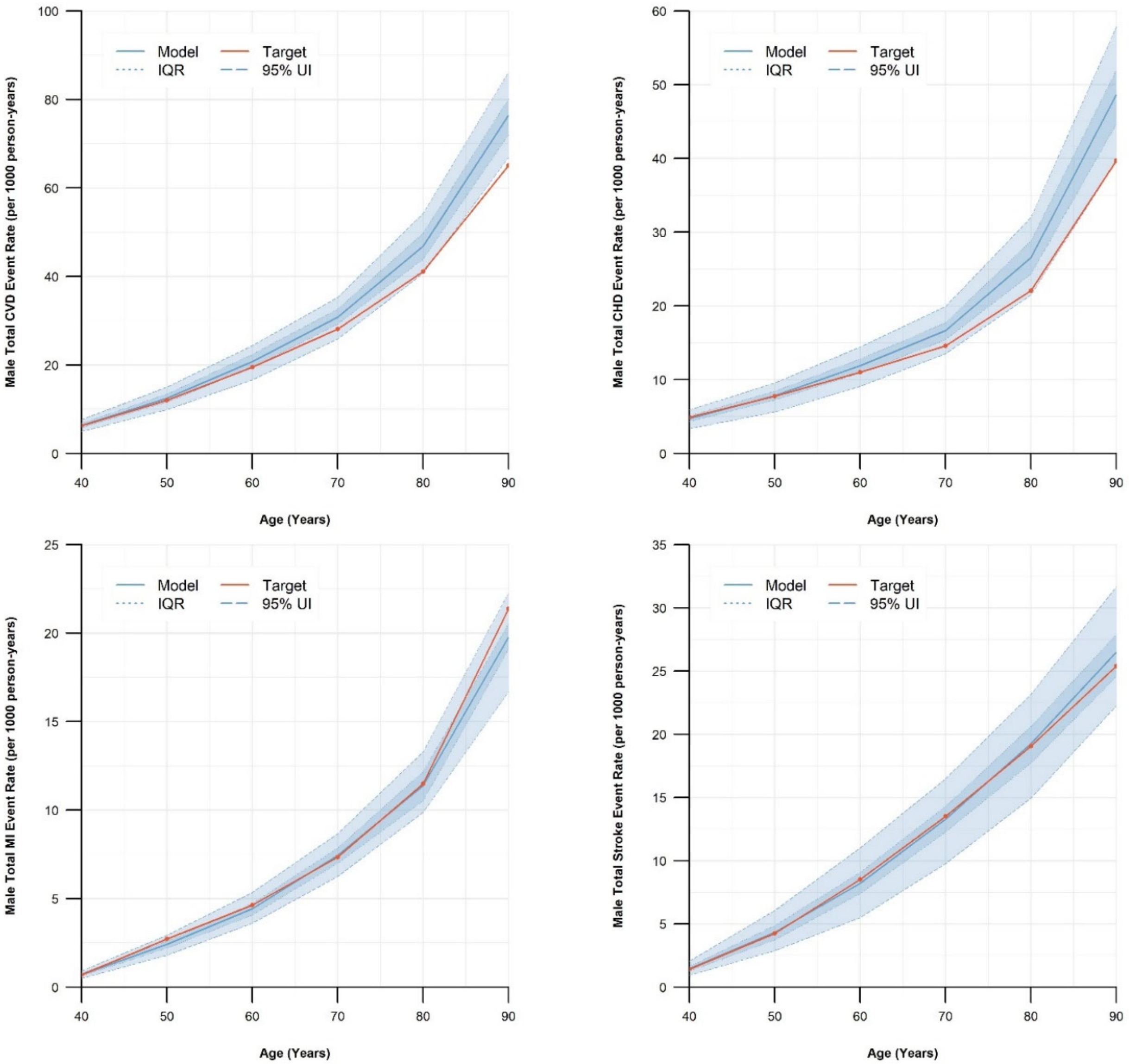

© 2025 Kohli-Lynch CN et al. *JAMA Cardiology*.

F. Recalibration/validation figures, men: incident event rates

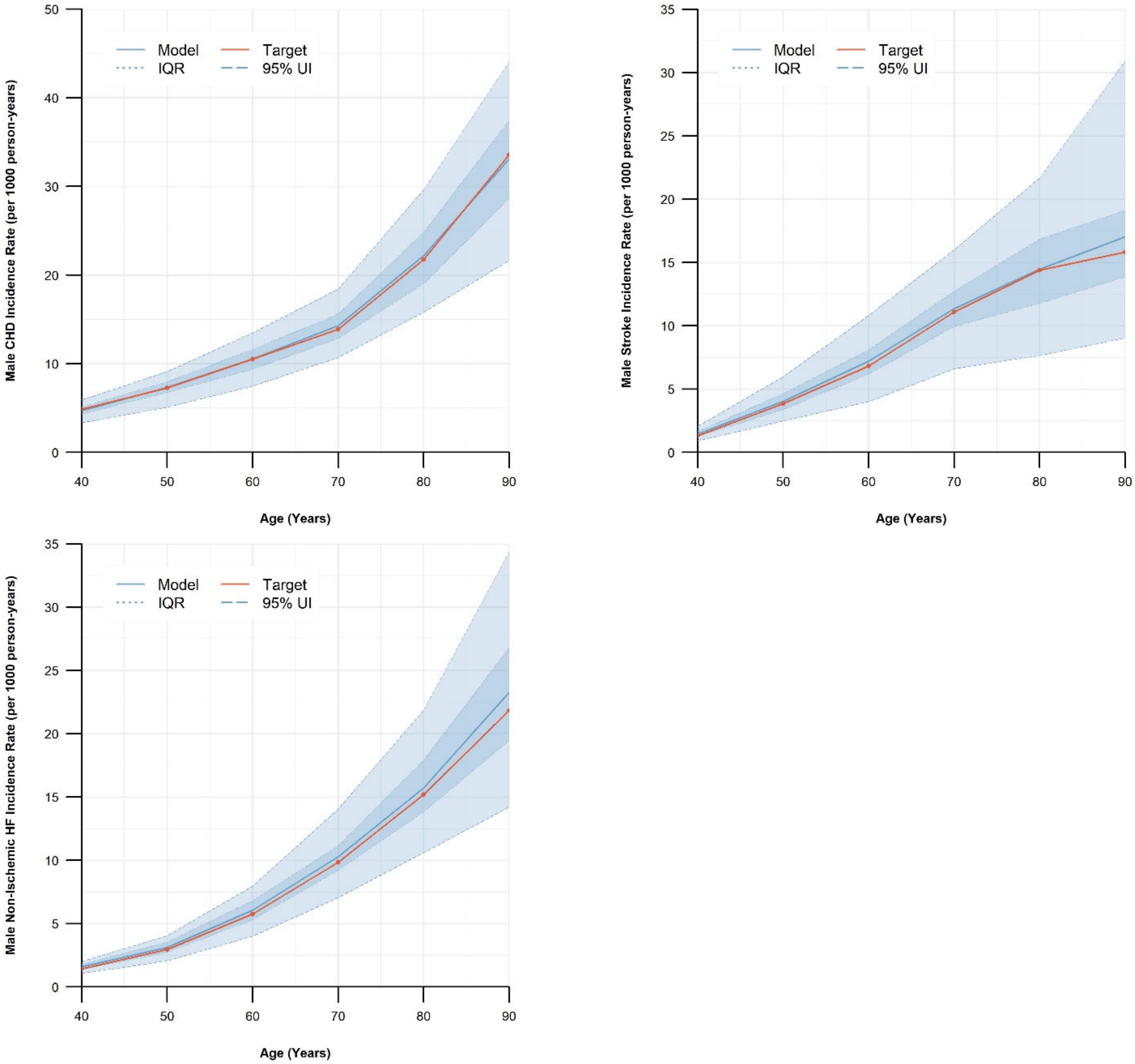

© 2025 Kohli-Lynch CN et al. *JAMA Cardiology*.

eFigure 4. Calibrating treatment effect

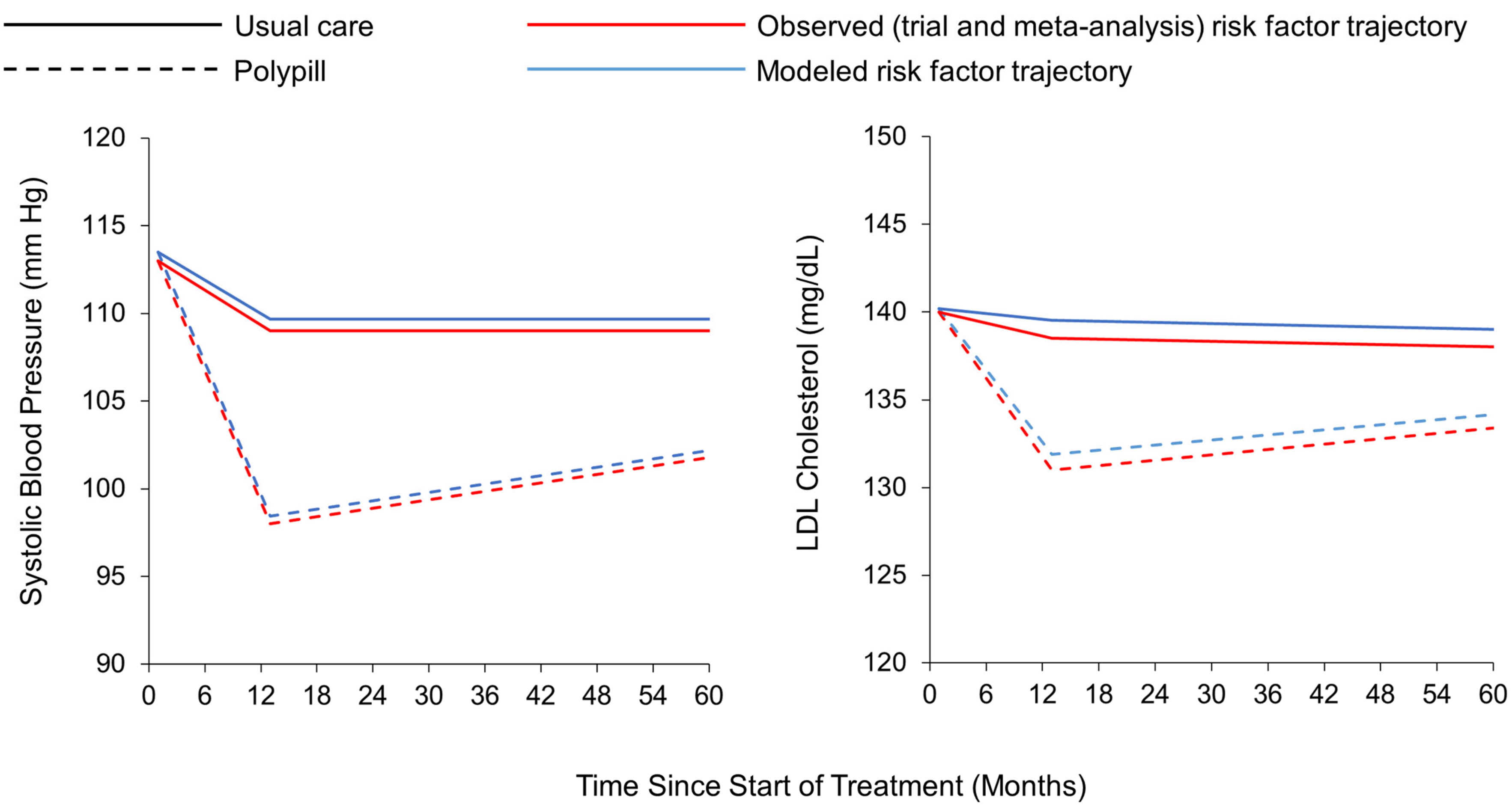

LDL – Low-Density Lipoprotein

© 2025 Kohli-Lynch CN et al. *JAMA Cardiology*.

eFigure 5. Modeled cost-effectiveness outcomes with different numbers of simulated individuals

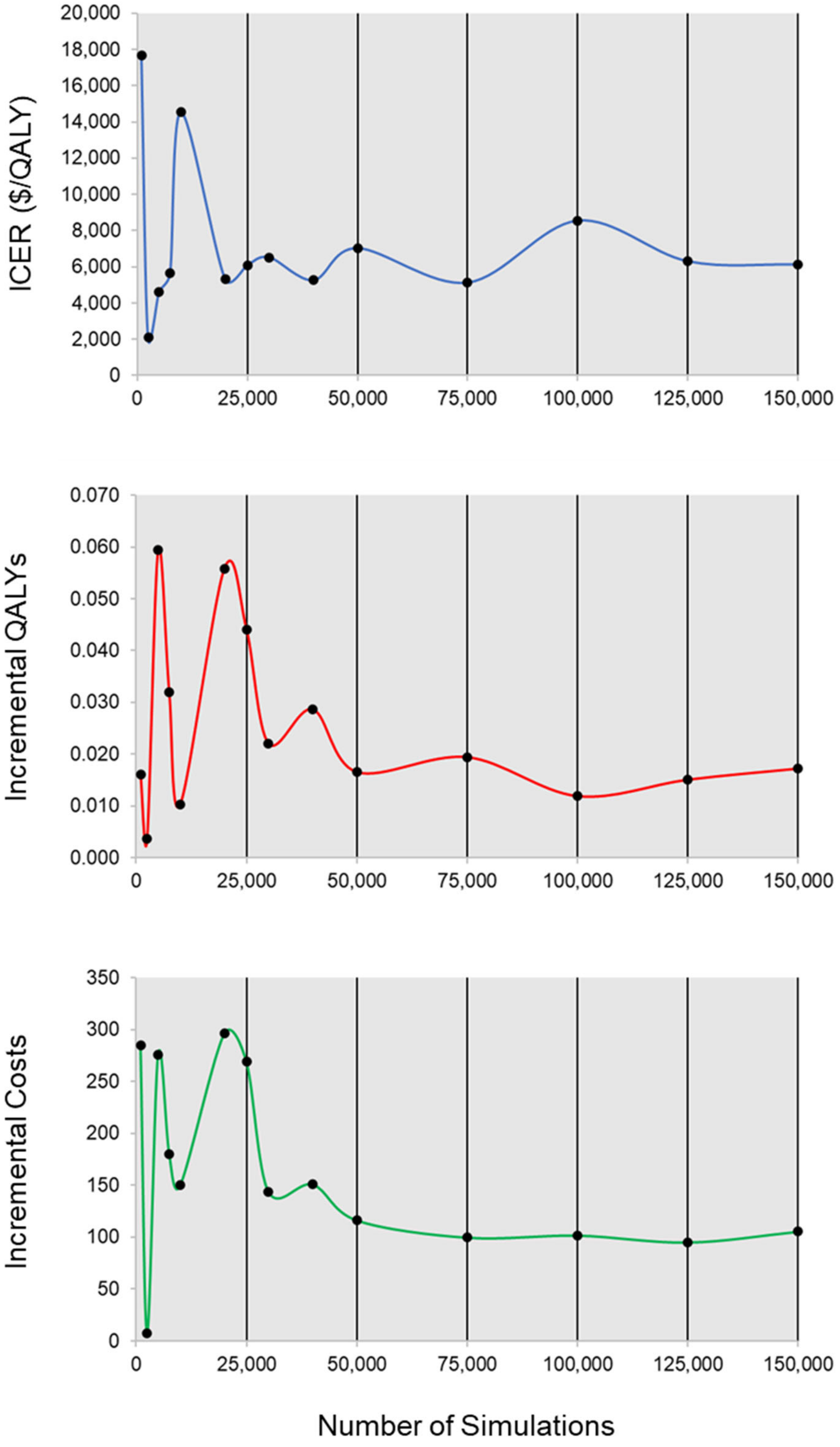

ICER – Incremental Cost-Effectiveness Ratio, QALY – Quality-Adjusted Life Year

Each simulation used same 'random seed'

© 2025 Kohli-Lynch CN et al. *JAMA Cardiology*.

eFigure 6. Cost-effectiveness acceptability curve (polypill trial population)

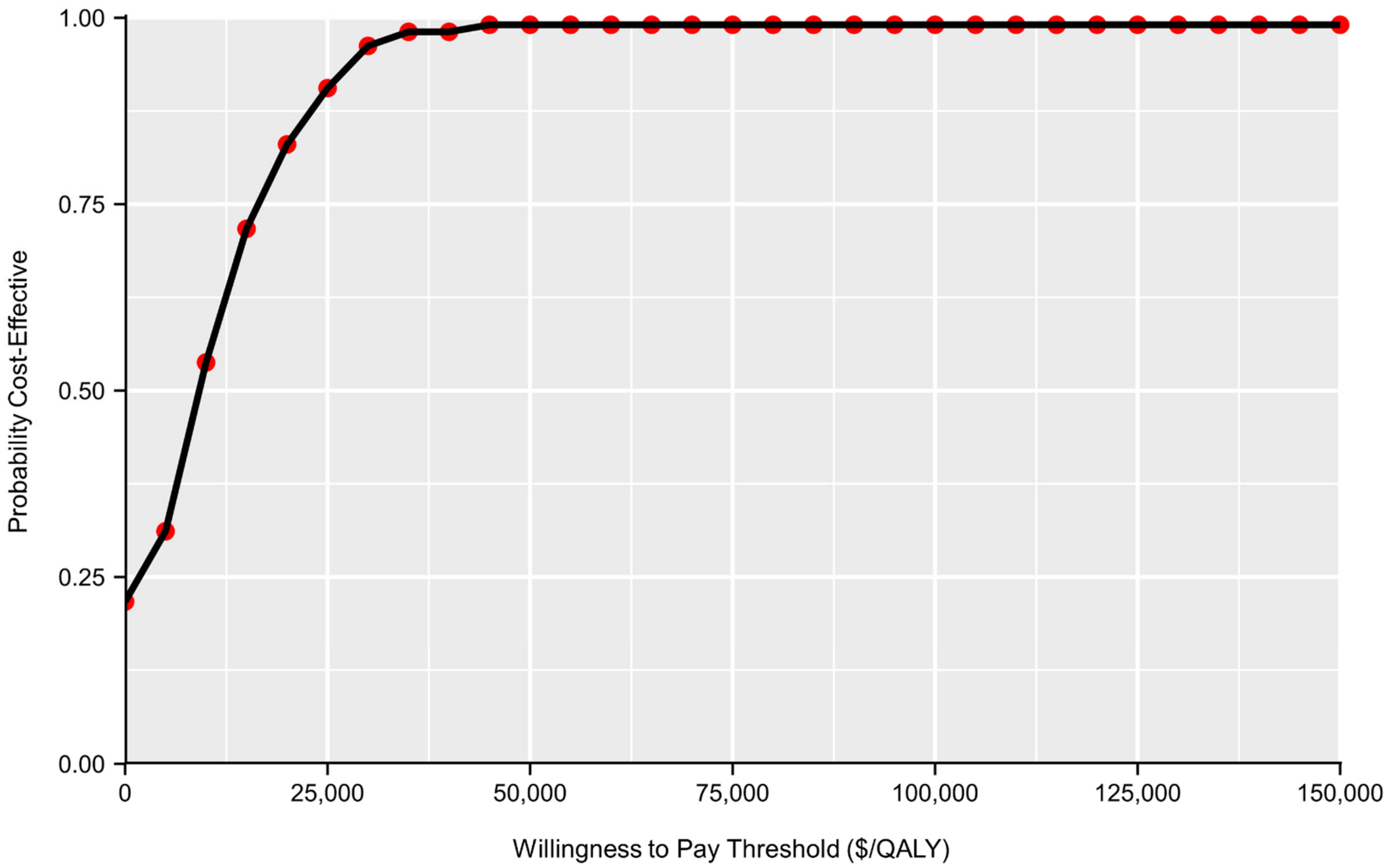

QALY – Quality-Adjusted Life Year

© 2025 Kohli-Lynch CN et al. *JAMA Cardiology*.

eFigure 7. Tornado diagram

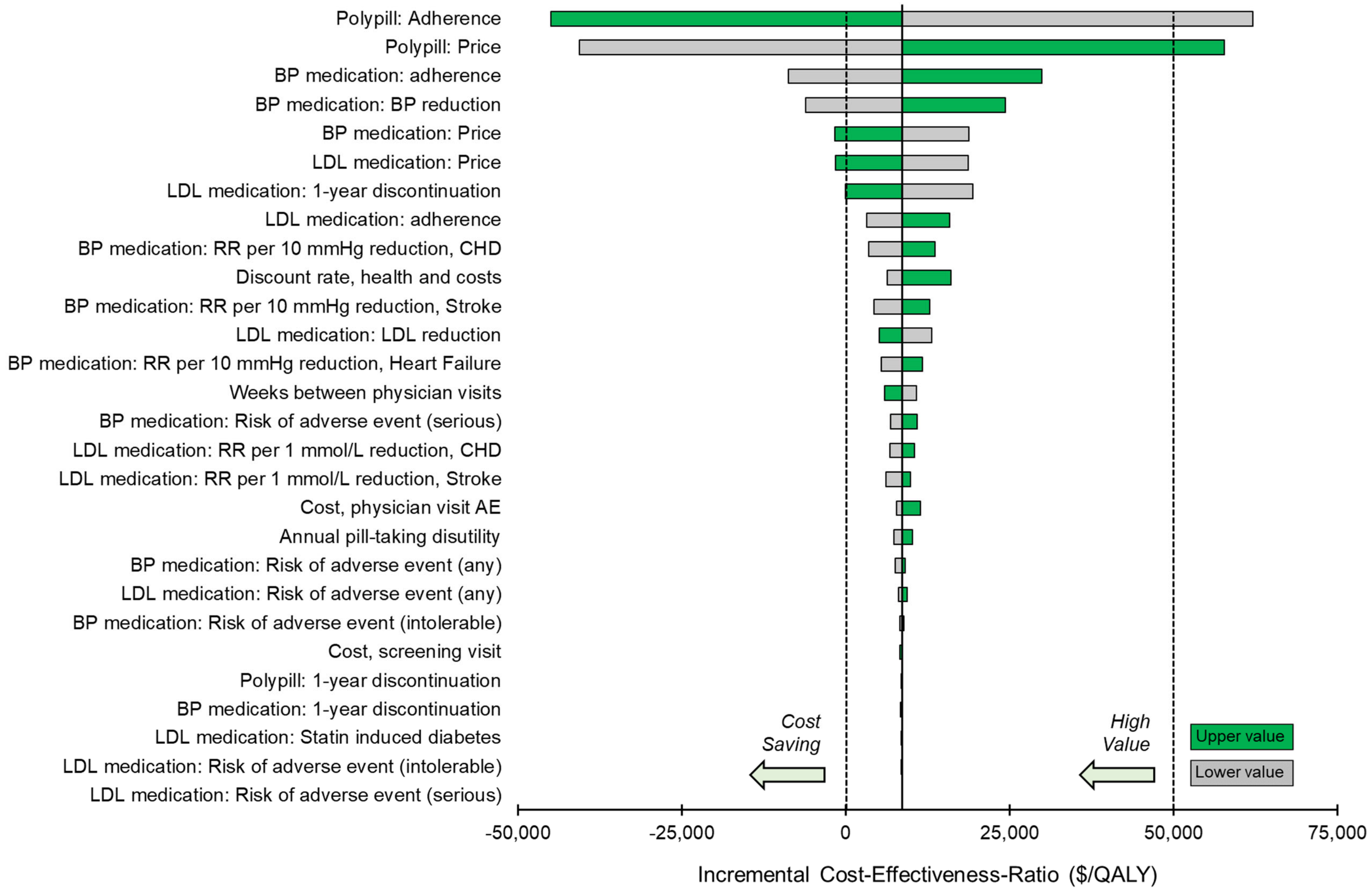

AE – Adverse Event, BP – Blood Pressure, CHD – Coronary Heart Disease, LDL – Low-Density Lipoprotein Cholesterol, QALY – Quality-Adjusted Life Year, RR – Relative Risk

No sensitivity analyses resulted in polypill being dominated (i.e., fewer QALYs, more costs), so all negative ICERs denote cost savings

© 2025 Kohli-Lynch CN et al. *JAMA Cardiology*.

eFigure 8. Incremental cost-effectiveness ratio for polypill versus usual care at range of time horizons

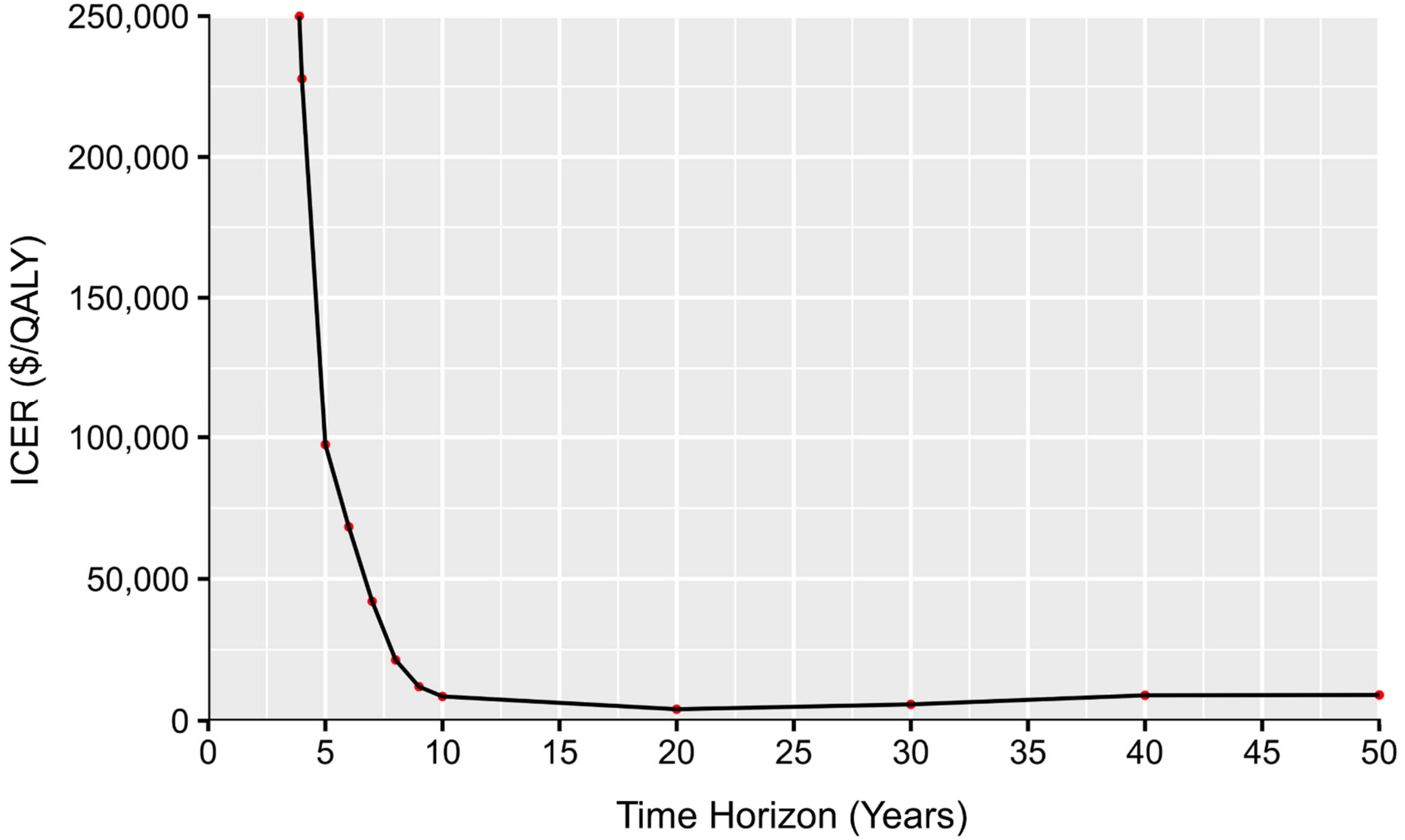

ICER – Incremental Cost-Effectiveness Ratio, QALY – Quality-Adjusted Life Year

Polypill 'dominated' (more costs, less QALYs) with 1-year and 2-year time horizons.
ICER=$424,000/QALY at 3-year time horizon

© 2025 Kohli-Lynch CN et al. *JAMA Cardiology*.

eFigure 9. Polypill price vs. ICER, polypill trial-eligible Non-Hispanic Black individuals

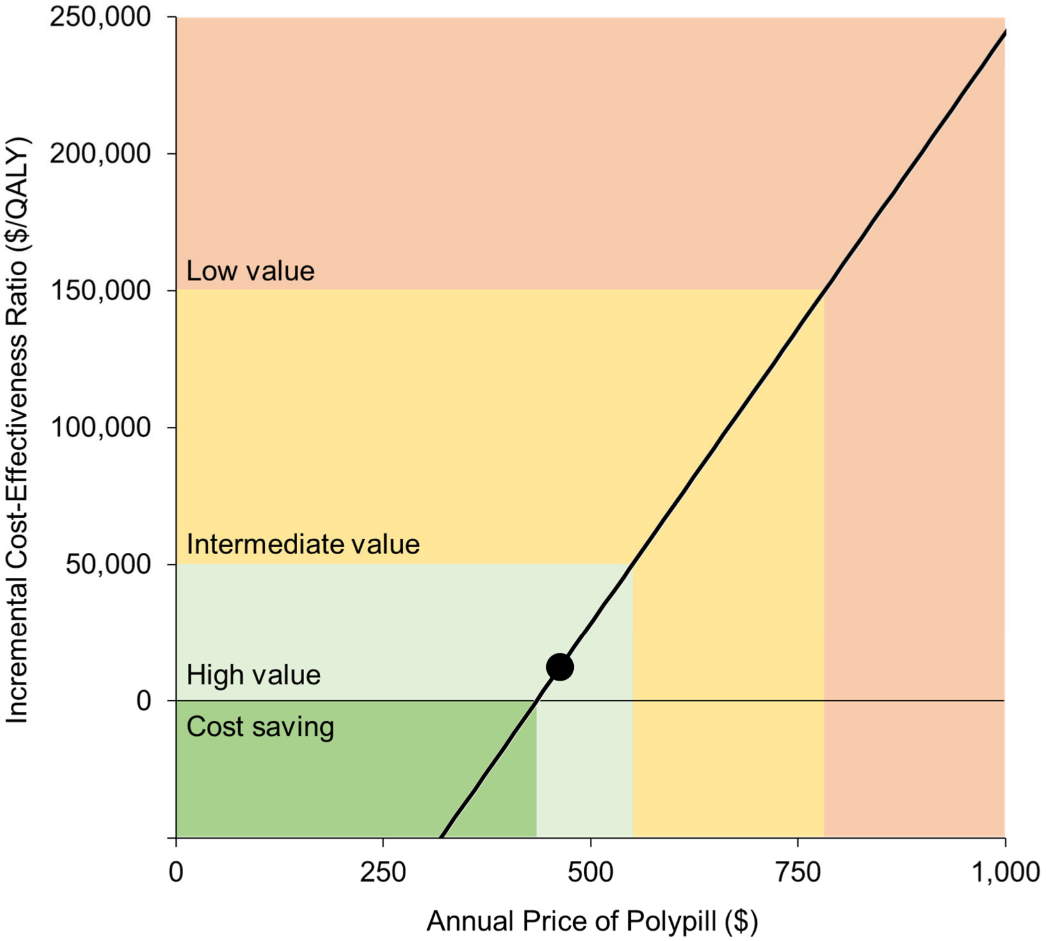

QALY – Quality-Adjusted Life Year

Black circle marks base case cost-effectiveness estimate.

© 2025 Kohli-Lynch CN et al. *JAMA Cardiology*.

eReferences

1. Kohli-Lynch CN, Bellows BK, Thanassoulis G, et al. Cost-effectiveness of Low-density Lipoprotein Cholesterol Level-Guided Statin Treatment in Patients With Borderline Cardiovascular Risk. *JAMA Cardiol*. 2019;4(10):969-977. doi:10.1001/jamacardio.2019.2851
2. Bryant KB, Moran AE, Kazi DS, et al. Cost-Effectiveness of Hypertension Treatment by Pharmacists in Black Barbershops. *Circulation*. 2021;143(24):2384-2394. doi:10.1161/CIRCULATIONAHA.120.051683
3. Bellows BK, Ruiz-Negrón N, Bibbins-Domingo K, et al. Clinic-Based Strategies to Reach United States Million Hearts 2022 Blood Pressure Control Goals. *Circulation: Cardiovascular Quality and Outcomes*. 2019;12(6):e005624. doi:10.1161/CIRCOUTCOMES.118.005624
4. Zhang Y, Vittinghoff E, Pletcher MJ, et al. Associations of Blood Pressure and Cholesterol Levels During Young Adulthood With Later Cardiovascular Events. *Journal of the American College of Cardiology*. 2019;74(3):330-341. doi:10.1016/j.jacc.2019.03.529
5. Oelsner EC, Balte PP, Cassano P, et al. Harmonization of Respiratory Data From Nine US Population-Based Cohorts: The NHLBI Pooled Cohorts Study. *Am J Epidemiol*. Published online June 30, 2018. doi:10.1093/aje/kwy139
6. Bryan AS, Moran AE, Mobley CM, et al. Cost-effectiveness analysis of initial treatment with single-pill combination antihypertensive medications. *J Hum Hypertens*. 2023;37(11):985-992. doi:10.1038/s41371-023-00811-3
7. Bryant KB, Rao AS, Cohen LP, et al. Effectiveness and Cost-Effectiveness of Team-Based Care for Hypertension: A Meta-Analysis and Simulation Study. *Hypertension*. 2023;80(6):1199-1208. doi:10.1161/HYPERTENSIONAHA.122.20292
8. Kohli-Lynch CN, Bellows BK, Zhang Y, et al. Cost-Effectiveness of Lipid-Lowering Treatments in Young Adults. *Journal of the American College of Cardiology*. 2021;78(20):1954-1964. doi:10.1016/j.jacc.2021.08.065
9. Ridgeway G, McCaffrey DF, Morral AR, et al. *Toolkit for Weighting and Analysis of Nonequivalent Groups: A Tutorial for the R TWANG Package*. RAND Corporation; 2022. Accessed July 13, 2023. https://www.rand.org/pubs/tools/TLA570-5.html
10. Ettehad D, Emdin CA, Kiran A, et al. Blood pressure lowering for prevention of cardiovascular disease and death: a systematic review and meta-analysis. *The Lancet*. 2016;387(10022):957-967. doi:10.1016/S0140-6736(15)01225-8
11. Mihaylova B, Emberson J, Blackwell L, et al. The effects of lowering LDL cholesterol with statin therapy in people at low risk of vascular disease: meta-analysis of individual data from 27 randomised trials. *Lancet*. 2012;380(9841):581-590 p 588. doi:10.1016/S0140-6736(12)60367-5
12. Law M, Wald N, Morris J. Lowering blood pressure to prevent myocardial infarction and stroke: a new preventive strategy. In: *NIHR Health Technology Assessment Programme: Executive Summaries*. NIHR Journals Library; 2003. Accessed July 6, 2023. https://www.ncbi.nlm.nih.gov/books/NBK62259/
13. Law MR, Wald NJ, Morris JK, Jordan RE. Value of low dose combination treatment with blood pressure lowering drugs: analysis of 354 randomised trials. *BMJ*. 2003;326(7404):1427. doi:10.1136/bmj.326.7404.1427
14. SPRINT Research Group, Wright JT, Williamson JD, et al. A Randomized Trial of Intensive versus Standard Blood-Pressure Control. *N Engl J Med*. 2015;373(22):2103-2116. doi:10.1056/NEJMoa1511939

© 2025 Kohli-Lynch CN et al. *JAMA Cardiology*.

15. Xie X, Atkins E, Lv J, et al. Effects of intensive blood pressure lowering on cardiovascular and renal outcomes: updated systematic review and meta-analysis. *Lancet*. 2016;387(10017):435-443. doi:10.1016/S0140-6736(15)00805-3

16. Cai T, Abel L, Langford O, et al. Associations between statins and adverse events in primary prevention of cardiovascular disease: systematic review with pairwise, network, and dose-response meta-analyses. *BMJ*. 2021;374:n1537. doi:10.1136/bmj.n1537

17. Sattar N, Preiss D, Murray HM, et al. Statins and risk of incident diabetes: a collaborative meta-analysis of randomised statin trials. *Lancet*. 2010;375(9716):735-742 p 741. doi:10.1016/S0140-6736(09)61965-6

18. Bryant KB, Jannat-Khah DP, Cornelius T, et al. Time-Varying Depressive Symptoms and Cardiovascular and All-Cause Mortality: Does the Risk Vary by Age or Sex? *Journal of the American Heart Association*. 2020;9(19):e016661. doi:10.1161/JAHA.120.016661

19. Kronish IM, Edmondson D, Shimbo D, Shaffer JA, Krakoff LR, Schwartz JE. A Comparison of the Diagnostic Accuracy of Common Office Blood Pressure Measurement Protocols. *Am J Hypertens*. 2018;31(7):827-834. doi:10.1093/ajh/hpy053

20. Fontil V, Bibbins-Domingo K, Kazi DS, et al. Simulating Strategies for Improving Control of Hypertension Among Patients with Usual Source of Care in the United States: The Blood Pressure Control Model. *J Gen Intern Med*. 2015;30(8):1147-1155. doi:10.1007/s11606-015-3231-8

21. Lin I, Sung J, Sanchez RJ, et al. Patterns of Statin Use in a Real-World Population of Patients at High Cardiovascular Risk. *J Manag Care Spec Pharm*. 2016;22(6):685-698. doi:10.18553/jmcp.2016.22.6.685

22. Vrijens B, Vincze G, Kristanto P, Urquhart J, Burnier M. Adherence to prescribed antihypertensive drug treatments: longitudinal study of electronically compiled dosing histories. *BMJ*. 2008;336(7653):1114-1117. doi:10.1136/bmj.39553.670231.25

23. Wood F, Salam A, Singh K, et al. Process evaluation of the impact and acceptability of a polypill for prevention of cardiovascular disease. *BMJ Open*. 2015;5(9):e008018. doi:10.1136/bmjopen-2015-008018

24. Muñoz D, Uzoije P, Reynolds C, et al. Polypill for Cardiovascular Disease Prevention in an Underserved Population. *N Engl J Med*. 2019;381(12):1114-1123. doi:10.1056/NEJMoa1815359

25. Claxton AJ, Cramer J, Pierce C. A systematic review of the associations between dose regimens and medication compliance. *Clin Ther*. 2001;23(8):1296-1310. doi:10.1016/s0149-2918(01)80109-0

26. Bryant KB, Sheppard JP, Ruiz-Negrón N, et al. Impact of Self-Monitoring of Blood Pressure on Processes of Hypertension Care and Long-Term Blood Pressure Control. *J Am Heart Assoc*. 2020;9(15):e016174. doi:10.1161/JAHA.120.016174

27. Law MR, Morris JK, Wald NJ. Use of blood pressure lowering drugs in the prevention of cardiovascular disease: meta-analysis of 147 randomised trials in the context of expectations from prospective epidemiological studies. *BMJ*. 2009;338:b1665. doi:10.1136/bmj.b1665

28. Lowy A, Munk VC, Ong SH, et al. Effects on blood pressure and cardiovascular risk of variations in patients' adherence to prescribed antihypertensive drugs: role of duration of drug action. *Int J Clin Pract*. 2011;65(1):41-53. doi:10.1111/j.1742-1241.2010.02569.x

29. Adams SP, Tsang M, Wright JM. Atorvastatin for lowering lipids. *Cochrane Database Syst Rev*. 2015;2015(3):CD008226. doi:10.1002/14651858.CD008226.pub3

30. *Consumer Price Index Databases*. U.S. Bureau of Labor Statistics; 2023. Accessed February 27, 2023. https://www.bls.gov/cpi/data.htm

31. *Medical Expenditure Panel Survey 2020*.; 2020. http://meps.ahrq.gov/mepsweb/

© 2025 Kohli-Lynch CN et al. *JAMA Cardiology*.

32. Harris-Kojetin L, Sengupta M, Park-Lee E, et al. Long-Term Care Providers and services users in the United States: data from the National Study of Long-Term Care Providers, 2013-2014. *Vital Health Stat 3*. 2016;(38):x-xii; 1-105.

33. Howden L, Meyer J. *Age and Sex Composition: 2010*. U.S. Department of Commerce Economics and Statistics Administration: U.S. Census Bureau; 2011. https://www.census.gov/library/publications/2011/dec/c2010br-03.html

34. *Affordable Care Act – Federal Upper Limit*. Centers for Medicare and Medicaid Services; 2023. Accessed January 4, 2024. https://www.medicaid.gov/medicaid/prescription-drugs/federal-upper-limit/index.html

35. *NADAC (National Average Drug Acquisition Cost)*. Centers for Medicare and Medicaid Services; 2023. Accessed January 5, 2024. https://data.medicaid.gov/nadac

36. *Medicaid Covered Outpatient Prescription Drug Reimbursement Information by State*. Centers for Medicare and Medicaid Services; 2023. Accessed January 5, 2024. https://www.medicaid.gov/medicaid/prescription-drugs/state-prescription-drug-resources/medicaid-covered-outpatient-prescription-drug-reimbursement-information-state/index.html

37. *National Inpatient Sample 2012-2015*. Agency for Healthcare Research and Quality; 2019. https://hcup-us.ahrq.gov/nisoverview.jsp

38. Arth AC. Inpatient Hospitalization Costs Associated with Birth Defects Among Persons of All Ages — United States, 2013. *MMWR Morb Mortal Wkly Rep*. 2017;66. doi:10.15585/mmwr.mm6602a1

39. Peterson C, Xu L, Florence C, Grosse SD, Annest JL. Professional Fee Ratios for US Hospital Discharge Data. *Med Care*. 2015;53(10):840-849. doi:10.1097/MLR.0000000000000410

40. Moran AE, Forouzanfar MH, Roth GA, et al. Temporal trends in ischemic heart disease mortality in 21 world regions, 1980 to 2010: the Global Burden of Disease 2010 study. *Circulation*. 2014;129(14):1483-1492. doi:10.1161/CIRCULATIONAHA.113.004042

41. Moran AE, Forouzanfar MH, Roth GA, et al. The global burden of ischemic heart disease in 1990 and 2010: the Global Burden of Disease 2010 study. *Circulation*. 2014;129(14):1493-1501. doi:10.1161/CIRCULATIONAHA.113.004046

42. Murray CJL, Vos T, Lozano R, et al. Disability-adjusted life years (DALYs) for 291 diseases and injuries in 21 regions, 1990–2010: a systematic analysis for the Global Burden of Disease Study 2010. *The Lancet*. 2012;380(9859):2197-2223. doi:10.1016/S0140-6736(12)61689-4

43. Joseph P, Roshandel G, Gao P, et al. Fixed-dose combination therapies with and without aspirin for primary prevention of cardiovascular disease: an individual participant data meta-analysis. *The Lancet*. 2021;398(10306):1133-1146. doi:10.1016/S0140-6736(21)01827-4

44. McManus DD, Chinali M, Saczynski JS, et al. Thirty-Year Trends in Heart Failure in Patients Hospitalized With Acute Myocardial Infarction. *Am J Cardiol*. 2011;107(3):353-359. doi:10.1016/j.amjcard.2010.09.026

45. Spencer FA, Meyer TE, Gore JM, Goldberg RJ. Heterogeneity in the Management and Outcomes of Patients With Acute Myocardial Infarction Complicated by Heart Failure. *Circulation*. 2002;105(22):2605-2610. doi:10.1161/01.CIR.0000017861.00991.2F

46. Hernandez GA, Lemor A, Blumer V, et al. Trends in Utilization and Outcomes of Pulmonary Artery Catheterization in Heart Failure With and Without Cardiogenic Shock. *J Card Fail*. 2019;25(5):364-371. doi:10.1016/j.cardfail.2019.03.004

47. Steg PG, Dabbous OH, Feldman LJ, et al. Determinants and prognostic impact of heart failure complicating acute coronary syndromes: observations from the Global Registry of Acute Coronary Events (GRACE). *Circulation*. 2004;109(4):494-499. doi:10.1161/01.CIR.0000109691.16944.DA

© 2025 Kohli-Lynch CN et al. *JAMA Cardiology*.

48. Gerber Y, Weston SA, Enriquez-Sarano M, et al. Mortality Associated With Heart Failure After Myocardial Infarction: A Contemporary Community Perspective. *Circ Heart Fail*. 2016;9(1):e002460. doi:10.1161/CIRCHEARTFAILURE.115.002460

49. Benjamin EJ, Muntner P, Alonso A, et al. Heart Disease and Stroke Statistics-2019 Update: A Report From the American Heart Association. *Circulation*. 2019;139(10):e56-e528. doi:10.1161/CIR.0000000000000659

50. *Healthcare Cost and Utilization Project (HCUP)*. Agency for Healthcare Research and Quality; 2006. www.hcup-us.ahrq.gov/databases.jsp

51. Krumholz HM, Normand SLT, Wang Y. Trends in hospitalizations and outcomes for acute cardiovascular disease and stroke, 1999-2011. *Circulation*. 2014;130(12):966-975. doi:10.1161/CIRCULATIONAHA.113.007787

52. Centers for Medicare and Medicaid Services: Physician Fee Schedule. Accessed December 7, 2023. https://www.cms.gov/medicare/payment/fee-schedules/physician

53. *Unites States Renal Data System Annual Data Report 2020*. National Institutes of Health, National Institute of Diabetes and Digestive and Kidney Diseases; 2020. Accessed February 27, 2023. https://adr.usrds.org/

54. Bress AP, Bellows BK, King JB, et al. Cost-Effectiveness of Intensive versus Standard Blood-Pressure Control. *New England Journal of Medicine*. 2017;377(8):745-755. doi:10.1056/NEJMsa1616035

55. King JB, Shah RU, Bress AP, Nelson RE, Bellows BK. Cost-Effectiveness of Sacubitril-Valsartan Combination Therapy Compared With Enalapril for the Treatment of Heart Failure With Reduced Ejection Fraction. *JACC Heart Fail*. 2016;4(5):392-402. doi:10.1016/j.jchf.2016.02.007

56. Sullivan PW, Ghushchyan V. Preference-Based EQ-5D Index Scores for Chronic Conditions in the United States. *Med Decis Making*. 2006;26(4):410-420. doi:10.1177/0272989X06290495

57. Hutchins R, Pignone MP, Sheridan SL, Viera AJ. Quantifying the utility of taking pills for preventing adverse health outcomes: a cross-sectional survey. *BMJ Open*. 2015;5(5):e006505. doi:10.1136/bmjopen-2014-006505

58. Hutchins R, Viera AJ, Sheridan SL, Pignone MP. Quantifying the utility of taking pills for cardiovascular prevention. *Circ Cardiovasc Qual Outcomes*. 2015;8(2):155-163. doi:10.1161/CIRCOUTCOMES.114.001240

59. *National Health and Nutrition Examination Survey Data. Centers for Disease Control and Prevention. National Center for Health Statistics.* U.S. Department of Health and Human Services. Centers for Disease Control and Prevention; 1999. https://wwwn.cdc.gov/Nchs/Nhanes/

60. Newman CB, Preiss D, Tobert JA, et al. Statin Safety and Associated Adverse Events: A Scientific Statement From the American Heart Association. *Arterioscler Thromb Vasc Biol*. 2019;39(2):e38-e81. doi:10.1161/ATV.0000000000000073

61. Finegold JA, Manisty CH, Goldacre B, Barron AJ, Francis DP. What proportion of symptomatic side effects in patients taking statins are genuinely caused by the drug? Systematic review of randomized placebo-controlled trials to aid individual patient choice. *Eur J Prev Cardiolog*. 2014;21(4):464-474. doi:10.1177/2047487314525531

62. Husereau D, Drummond M, Augustovski F, et al. Consolidated Health Economic Evaluation Reporting Standards 2022 (CHEERS 2022) statement: updated reporting guidance for health economic evaluations. *BMC Medicine*. 2022;20(1):23. doi:10.1186/s12916-021-02204-0

© 2025 Kohli-Lynch CN et al. *JAMA Cardiology*.
