# Supplementary material for: Cost-Effectiveness of a Polypill for Cardiovascular Disease Prevention in an Underserved Population
Source: JAMA Cardiol. 2025 Jan 8;10(3):224–33. doi: 10.1001/jamacardio.2024.4812 (PMC11904704; doi:10.1001/jamacardio.2024.4812)
Supplement: Supplement 2. — Data Sharing Statement. [file jamacardiol-e244812-s002.pdf]

Data Sharing Statement

Kohli-Lynch. Cost-Effectiveness of a Polypill for Cardiovascular Disease Prevention in an Underserved Population. *JAMA Cardiol*. Published January 08, 2025. doi:10.1001/jamacardio.2024.4812

Data
Data available: No

Additional Information
Explanation for why data not available: The results of our research are derived from a decision-analytic model that is not open source. However, the model was constructed using deidentified or publicly available data. While the raw data used in the model can be accessed publicly or upon request from the respective data custodians, the specific decision-analytic model itself cannot be shared due to proprietary restrictions.
